# ^90^Y-/^166^Ho- ‘Radiation lobectomy’ for liver tumors induces abnormal morphology and impaired drainage of peritumor lymphatics

**DOI:** 10.1016/j.jhepr.2023.100981

**Published:** 2023-12-05

**Authors:** Daan Andel, Lotte van den Bent, Marnix Gerard Ernest Hendrik Lam, Maarten Leonard Johannes Smits, Isaac Quintus Molenaar, Joep de Bruijne, Miangela Marie Laclé, Onno Kranenburg, Inne Hildbrand Max Borel Rinkes, Jeroen Hagendoorn

**Affiliations:** 1 Department of Surgical Oncology, University Medical Center Utrecht, Cancer Center, Utrecht, The Netherlands; 2 Laboratory for Translational Oncology, University Medical Center Utrecht, Cancer Center, Utrecht, The Netherlands; 3 Department of Radiology and Nuclear Medicine, University Medical Center Utrecht, Cancer Center, Utrecht, The Netherlands; 4 Department Gastroenterology and Hepatology, University Medical Center Utrecht, Utrecht, the Netherlands; 5 Department of Pathology, University Medical Center Utrecht, Utrecht University, Utrecht, The Netherlands

**Keywords:** Lymphatics, Radiation lobectomy, Radioembolization, Intraoperative liver lymphangiography, hcc, crlm, Immunohistochemistry

## Abstract

**Background & Aims:**

High-dose unilobar radioembolization, or ‘radiation lobectomy’ (RL), is an induction therapy that achieves contralateral future liver remnant hypertrophy while simultaneously irradiating the tumor. As such, it may prevent further growth, but it is unknown whether RL affects intrahepatic lymphatics, a major route via which liver tumors disseminate.

**Methods:**

This was a case-control study conducted at University Medical Center Utrecht. The study compared lymph vessels in livers that had undergone RL (cases) with those in livers that had not undergone RL (controls). Histological samples were acquired from patients diagnosed with hepatocellular carcinoma (HCC) or colorectal liver metastases (CRLM) between 2017 and 2022. Lymph vessel morphology was analyzed by two researchers using podoplanin, a protein that is expressed in lymphatic endothelium. *In vivo* liver lymph drainage of radioembolized livers was assessed using intraoperative liver lymphangiography (ILL): during liver surgery, patent blue dye was injected into the liver parenchyma, followed by inspection for staining of perihepatic lymph structures. ILL results were compared to a previously published cohort.

**Results:**

Immunohistochemical analysis on post-RL tumor tissues from ten patients with CRLM and nine patients with HCC revealed aberrant morphology of irradiated liver lymphatics when compared to controls (n = 3 per group). Irradiated lymphatics were tortuous (*p* <0.05), thickened (*p* <0.05) and discontinuous (*p* <0.05). Moreover, post-RL lymphatics had larger lumens (1.5–1.7x, *p* <0.0001), indicating lymph stasis. ILL revealed diminished lymphatic drainage to perihepatic lymph nodes and vessels in irradiated livers when compared to non-radioembolized controls (*p* = 1.0x10^-4^).

**Conclusions:**

Radioembolization impairs peritumoral lymph vessel function. Further research is needed to evaluate if radioembolization impairs tumor dissemination via this route.

**Impact and implications:**

Unilobar radioembolization can serve as an alternative to portal venous embolization for patients who are considered unresectable due to an insufficient future liver remnant. This research suggests that radioembolization impairs the function of peritumoral liver lymph vessels, potentially hindering dissemination via this route. These findings provide support for considering unilobar radioembolization over standard portal venous embolization.

## Highlights

• Peritumoral liver lymphatics are tortuous, thickened and discontinuous after radioembolization.
• Irradiated liver lymphatics are ectactic, indicating lymph stasis within the liver.
• *In vivo* lymphangiography reveals reduced lymph drainage from radioembolized liver lymphatics into perihilar lymph nodes and vessels.

## Graphical abstract

**Figure:**
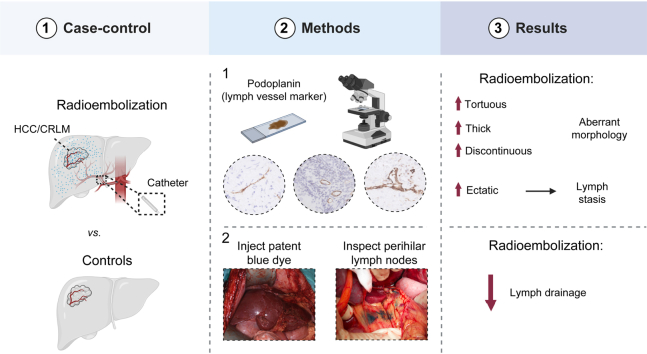

## Introduction

Radioembolization can be an effective treatment for primary and metastatic liver cancers.^1^^,^^2^ It uses the predominantly arterial blood supply of liver tumors to locally administrate b-radiation-emitting microspheres. For hepatocellular carcinoma (HCC), radioembolization has recently been added to the BCLC strategy for very early-to intermediate stage disease in patients who are not candidates for thermal ablation or resection.^3^ For colorectal liver metastases (CRLM), radioembolization is an option for patients with unresectable, refractory and liver-dominant disease.^2^ In both HCC and CRLM, radioembolization can be used as neoadjuvant treatment for patients that are deemed unresectable due to insufficiency of the future liver remnant.[4], [5], [6], [7] In these cases, high-dose, unilobar treatment may induce ipsilateral atrophy (thus termed ‘radiation lobectomy’ [RL]) and contralateral hypertrophy of the future liver remnant, while simultaneously treating the tumor.^5^

The main antitumor effects of radiation are attributed to DNA damage – either directly or through the formation of oxygen radicals,^8^ as well as the activity of the tumor microenvironment. Important determinants include activity of cancer-associated fibroblasts,^9^ modulation of the extracellular matrix,^10^ antitumor immunity^8^^,^^11^ as well as endothelial cell death and subsequent hypoxia.^12^ In addition, radiation causes apoptosis of lymphatic endothelial cells, loss of function of lymphatic vessels and subsequent lymphedema.^13^ All of these factors can influence tumor response, progression and dissemination through blood vessels and/or lymph vessels.

The pathophysiological importance of the liver lymphatic system is underscored by the large flux of liver lymph to the thoracic duct, which allows cells to spread to the lungs, and the frequent presence of metastases in hepatic pedicle lymph nodes.^14^^,^^15^ Moreover, the prognosis of patients with liver malignancies and metastases to the lymph nodes is worse than in those without lymph node involvement.[14], [15], [16] As endothelial cells are vulnerable to irradiation, the loss of lymphatic function induced by radioembolization could potentially hinder the dissemination of cancer cells through these routes.[16], [17], [18] Our group recently conducted two studies using liver lymphangiography, which demonstrated that lymph drainage follows the segmental anatomy of the liver.^19^^,^^20^ However, lymphatic function has not been characterized in radioembolized livers. In this study, we hypothesized that irradiated liver lymph vessels become dysfunctional. To evaluate this hypothesis, we analyzed lymphatic morphology in post-radioembolization tumor tissues and assessed lymph vessel function through intraoperative liver lymphangiography.

## Patients and methods

### Study setup and patient selection

This was a case-control study conducted at University Medical Center (UMC) Utrecht. The study aimed to compare lymph vessels in livers that had undergone RL (cases) with those in livers that had not undergone RL (controls). This comparison was conducted using two methods: (i) immunohistochemical (IHC) analysis to assess lymph vessel morphology and (ii) intraoperative liver lymphangiography (ILL) to evaluate liver lymphatic drainage to perihilar lymph nodes and lymph vessels.

For the IHC analyses, histological sections were obtained from patients with HCC or CRLM who received RL and hemihepatectomy between 2017 and 2022. Controls consisted of patients who underwent hemihepatectomy without prior RL and were selected from the surgical pathology files. Cases and controls were matched for tumor type, extent and size, age, preoperative chemotherapy status and metastatic lymph node involvement.

For the ILL analyses (refer to the 'Intraoperative liver lymphangiography' section for procedure details), we utilized the established framework from the LILY study. The LILY study's objective was to map the drainage patterns of the liver lymphatic system using patent blue dye in patients without a history of prior liver surgery or liver irradiation.^19^ ILL is routinely used during liver resection at UMC Utrecht to assess (potentially) tumor-draining lymph nodes. For the case group, we mapped draining patterns in all patients with CRLM or HCC who had undergone prior RL between 2022 and 2023 and for whom intraoperative liver lymphangiography was clinically indicated. Patients with a prior history of liver radiation, other than radioembolization, or prior liver surgery were not included in the study as cases. As controls, we reanalyzed the previously published data from the LILY cohort, focusing on patients with HCC or CRLM. Additionally, we mapped lymph drainage in two non-radioembolized patients with HCC. We extracted confounding factors that could potentially influence lymphatic drainage, such as cirrhosis, fibrosis, hepatitis, and steatosis from the patients' electronic health records and pathology files.

**Table 2:** Patient characteristics of the ILL cohort.

	ILL (n = 20)		*p* value§
	Control^1^ (n = 12)	RL (n = 8)	
Demographics			
Sex (M/F)	7/5	6/2	0.64
Tumor (CRLM/HCC)	9/3	4/4	0.17
Age (years)	66 [50-80]	67 [48-76]	0.91
Baseline characteristics			
Cirrhosis	1	0	1
Fibrosis	4	3	1
Hepatitis	3	3	1
Steatosis	4	5	0.37
Previous treatment			
Chemotherapy	5	4	1
PVE	4	1	0.60
Treatment details			
Microsphere (^90^Y/^166^Ho)	n.a.	3/5	n.a.
Time treatment to surgery (days)∗	56 ± 52	97 ± 25	0.17
Surgery			
Major/minor∗∗	8/4	8/0	0.12

^166^Ho, holmium-166, ^90^Y, yttrium-90; CRLM, colorectal liver metastases; HCC, hepatocellular carcinoma; ILL, intraoperative liver lymphangiography; PVE, portal vein embolization; RL, radiation lobectomy.

Demographics, baseline characteristics and treatment details. Age at time of surgery as median (range).

1 In part obtained from ‘van den Bent *et al.*, BJS, 2022’.

∗ The time from treatment to surgery indicates the time between the last treatment (RL in the case group, systemic treatment in the control group, if received) and hepatectomy.

∗∗ Major: (extended) hemihepatectomy; minor: wedge or segment resection(s).

§ Difference between radioembolized (RL) and non-radioembolized (Control) group. Fisher’s exact test and Mann-Whitney *U* test for categorical and numerical variables, respectively.

The need for written informed consent was waived by the UMC Utrecht Institutional Review Board (reference 21/625, 10.12.22). The study was performed in accordance with the Declaration of Helsinki.

### Radiation lobectomy

All patients underwent unilobar radioembolization with the intent of inducing concomitant ipsilateral atrophy and contralateral (future liver remnant) hypertrophy (RL setting). The procedure was performed by a board-certified interventional radiologist (MLJS) and nuclear physician (MGEHL). Patients had to be chemotherapy naïve for at least 4 weeks prior to RL. Patients underwent a work-up angiography for safety and treatment planning. During this angiography, the hepatic arterial anatomy was mapped, sources of extrahepatic shunting were identified and addressed, and a scout dose was administered at selected injection position(s). C-arm CT with contrast injection through the intra-arterial catheter was typically performed at the injection position of each scout dose. The scout dose consisted of technetium-99m macroalbumin aggregates (^99m^Tc-MAA) in patients treated with yttrium-90 (^90^Y) microspheres (Therasphere®, Boston Scientific) or holmium-166 (^166^Ho) scout (QuiremScout®, Terumo) in patients treated with ^166^Ho microspheres (QuiremSpheres®, Terumo). Work-up angiography was followed by SPECT-CT (single photon emission computed tomography) to determine the distribution of the scout dose. This information was used for safety (no excessive radiation to non-target structures) and for calculating the prescribed activity. The prescribed activity was calculated using the medical internal radiation dose mono-compartment method for ^90^Y glass and ^166^Ho microspheres, or the partition model in a selection of ^166^Ho-treated patients. The desired doses in the medical internal radiation dose model (MIRD) were 80–120 Gy for ^90^Y and 60 Gy for ^166^Ho.^21^ The actual RL treatment was performed no later than 2 weeks following the scout dose. The treatment dose was administered under angiography similar to the work-up angiography. All patients received unilobar treatment with or without segmentectomy. Segmentectomy was performed by administering microspheres into one of the segmental arteries. The dose depended on microsphere activity and tumor volume, but it typically exceeded 200 Gy. After RL, patients received follow-up imaging consisting of ^99m^Tc-mebrofenin hepatobiliary scintigraphy and CT or MRI scans at a 3-monthly interval to assess treatment effect and resectability.

### Surgery

The decision to resect was made after discussion in an hepato-pancreato-biliary (HPB) multidisciplinary tumor board. Patients were deemed candidates for resection in case of stable oncological disease. The future liver remnant was deemed sufficient for surgery if the function exceeded 2.69 %/min/m^2^ on hepatobiliary scintigraphy.^22^ The extent of the resection was primarily based on the segments embolized and the function of the liver remnant.

### Intraoperative liver lymphangiography

**Fig. 1:**
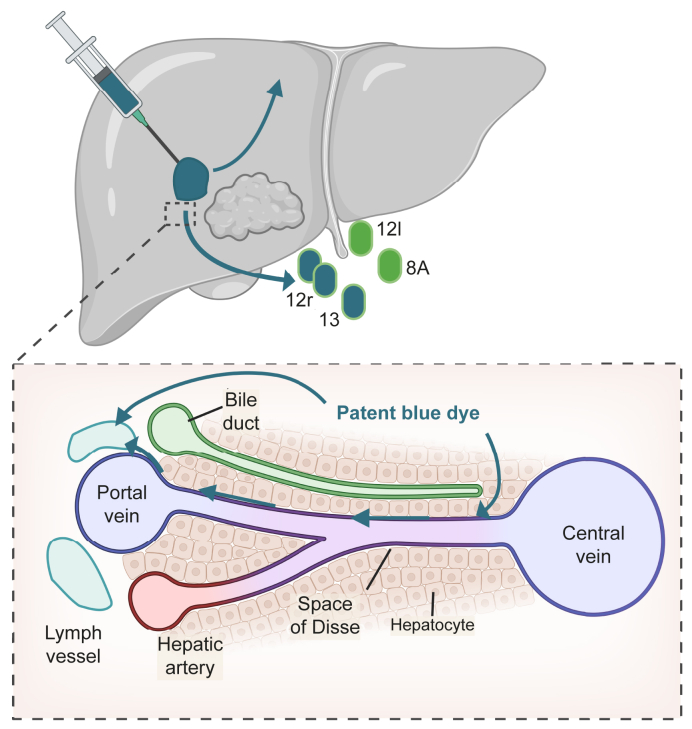
Schematic depicting the injection of patent blue dye and intrahepatic lymph flow. After injection, patent blue dye flows directly or via the Space of Disse into intrahepatic lymph vessels. The dye is then drained from these lymph vessels into extrahepatic lymph nodes and vessels located near the hepatoduodenal ligament, lesser omentum, and suprahepatic area toward the inferior vena cava. It is important to note that lymph vessels follow a segmental anatomy; for instance, the injection of patent blue dye into the right lobe will result in blue staining of nodes on the right side of the ligament, such as lymph stations 12r and 13 ^(^^19^^,^^20^^)^.

During the liver resection, ILL was performed by three board-certified HPB surgeons (IQM, JH, and IHMBR). Please refer to Fig. 1 (schematic) and Fig. S1 for a summary of the various steps involved in the lymphangiography procedure using patent blue dye. Patent blue dye is a high molecular weight protein tracer that is selectively absorbed by lymphatic vessels (Fig. 1). It is commonly used in clinical practice for lymph node mapping.

After entering the abdominal wall and visualizing the liver and tumor regions, 2 to 4 mL of patent blue dye were injected in 2 to 4 fractions (Fig. S1A). The dye was injected deep into the liver parenchyma, within two centimeters of the tumor, using a 15/20G needle. Subsequently, the liver was mobilized, and a waiting period of 5 to 10 min was allowed to permit the tracer to be absorbed by lymphatic vessels and transported to extraparenchymal lymph nodes and vessels. The examination for blue discoloration involved systematically inspecting perihepatic lymph vessels and lymph nodes, starting from the liver hilum and progressing towards the duodenum along the hepatoduodenal ligament (Fig. S1B). Following this, the minor omentum/gastrohepatic ligament was inspected (Fig. S1C), followed by the examination of lymph nodes along the suprahepatic inferior vena cava (Fig. S1D). This assessment included lymph nodes at stations 12r and 13 on the right side of the hepatoduodenal ligament, 12l on the left side of the hepatoduodenal ligament, 8A and 9 along the common hepatic artery, and station 7 in the minor omentum/gastrohepatic ligament (Fig. S2). Mediastinal nodes were inaccessible for direct observation.

An HPB surgeon filled out a case report form to document the injection site, the total volume of dye used, the time until the first staining of perihepatic lymph nodes or lymph vessels, and the exact anatomical location of stained lymph nodes and vessels (please see supplementary data file 1 for the case report form). Digital images were captured whenever feasible, and lymph nodes were excised as deemed appropriate by the surgeon.

### Immunohistochemistry

Stainings were performed on 4 μm thick formalin-fixed paraffin embedded sections mounted on a glass microscope slide. Formalin-fixed paraffin embedded sections were dewaxed and rehydrated, after which endogenous peroxidase was blocked with 1.5% hydrogen peroxide in phosphate-buffered saline. Heat-mediated antigen retrieval was performed in citrate buffer. Slides were incubated with a primary antibody for the lymphatic endothelial cell marker podoplanin (Sigma-Aldrich; HPA007534) overnight at 4 °C. The secondary antibody was incubated for 1 h at room temperature. 3,3′-diaminobenzidine was used as a chromogen and hematoxylin was used as a counterstain.

### Qualitative and quantitative analyses

Analyses of IHC data were performed by DA and LvdB, under the guidance of a board-certified pathologist (MML). Slides were digitized and peritumor regions were manually annotated using QuPath version 0.2.3. ‘Peritumor’ was defined as a 1 mm-thick region surrounding the tumor. Regions with extensive stroma were excluded because of false positive background staining of fibroblasts. For the qualitative analyses, a high-density peritumor region of 10 mm^2^ was selected for each tumor, wherein the percentage of tortuous, discontinuous, and thick-walled lymphatic vessels was calculated. Tortuosity was defined as the ratio of the length of the vessel wall to the longest diameter of the lymphatic vessel, corrected for the area of the lumen.^23^ For the quantitative analysis, each lymphatic vessel within the peritumor region was manually annotated, including the vessel lumen, to allow extraction of per vessel characteristics. Tumors in which no region could be found with at least 30 blood vessels were excluded (n = 4). Blinding was not possible in this study because of the frequent presence of microspheres in the pathology slides.

### Statistical analyses

Descriptive statistics are denoted as means ± standard deviation or medians (range) for age. Statistical analysis was performed in R version 4.2.0. Means (± standard error of the mean) were compared using the unpaired, two-tailed Student’s *t* test or Mann-Whitney *U* test, where appropriate. To compare lymphatic drainage between radioembolized and non-radioembolized patients, we used the Fisher’s exact test to determine if there was a significant association between previous RL and the presence or absence of patent blue dye staining in perihepatic lymph nodes or vessels.

## Results

### Patient characteristics

The IHC cohort (n = 19 total) included post-hepatectomy tumor tissues from three non-irradiated controls with CRLM and seven patients with CRLM who underwent RL (cases). Moreover, this cohort included three controls with HCC and six patients with HCC who underwent RL. Table 1 displays the patient demographics, baseline characteristics, and treatment details of the IHC cohort. In the CRLM group, two controls and seven cases received preoperative chemotherapy, while in the HCC group, all patients were chemonaive. Two patients with HCC had cirrhosis, one in the control group and one in the case group.

**Table 1:** Patient characteristics of the IHC cohort.

	IHC cohort (n = 19)			
	CRLM		HCC	
	Control (n = 3)	RL (n = 7)	Control (n = 3)	RL (n = 6)
Demographics				
Sex (M/F)	2/1	5/2	2/1	6/0
Age (years)	76 (64–83)	58 (46–78)	71 (71–76)	75 (63–79)
Baseline				
Cirrhosis	0	0	1	1
Previous chemo	2	7	0	0
Treatment details				
Microsphere (^90^Y/^166^Ho)	NA	5/2	n.a.	6/0
Time treatment to surgery (days)∗	23 and 61	118 ± 39	n.a.	167 ± 62
Surgery				
Major/minor∗∗	0/3	7/0	1/2	6/0

^166^Ho, holmium-166; ^90^Y, yttrium-90; CRLM, colorectal liver metastases; HCC, hepatocellular carcinoma; IHC, immunohistochemistry; RL, radiation lobectomy.

Demographics, baseline characteristics and treatment details of the immunohistochemistry cohort. Data are numbers, median (range) or mean ± standard deviation. Age at the time of liver surgery.

∗ The time from treatment to surgery indicates the time between the last treatment (RL in the case group, systemic treatment in the CRLM control group) and hepatectomy.

∗∗ Major: (extended) hemihepatectomy; minor: wedge or segment resection(s).

For the ILL cohort (n = 20 total), we reanalyzed ten non-radioembolized patients from a cohort previously published by our group and performed ILL on two additional controls in the present study (both HCC). Therefore, the control group consisted of nine patients with CRLM and three with HCC. In eight post-RL patients (cases), we performed ILL in the current study (four CRLM and four HCC). Table 2 provides detailed patient characteristics for the ILL cohort.

In the control (no RL) and case (RL) groups, five patients had evidence of cirrhosis/fibrosis and received preoperative chemotherapy (all CRLM), whereas four patients in the case group (all CRLM) received preoperative chemotherapy. The average time between the last cycle and surgery for those who received chemo in the control group was 56 ± 52 days, while the time between RL and surgery in the case group was 97 ± 25 days.

### Peritumor liver lymph vessels display abnormal morphology and signs of fluid stasis after radioembolization

**Fig. 2:**
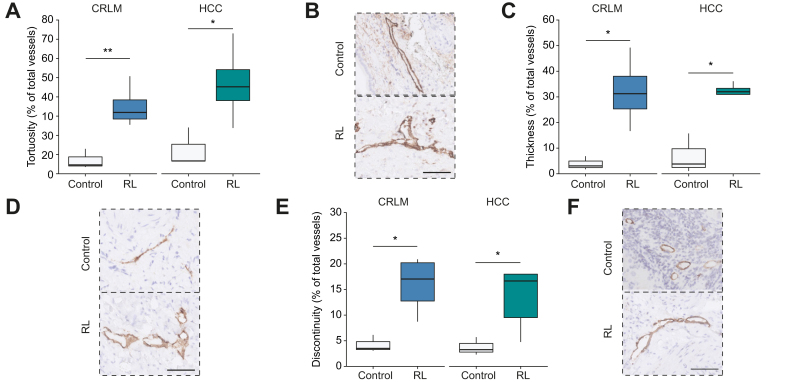
Peritumor lymphatics have aberrant morphology post-RL. (A) Comparison of percentages of tortuous lymphatics in control *vs.* post-RL specimens from patients with CRLM and HCC, and representative example in (B). (C) Frequencies of thickened walls in controls *vs.* post-RL, and example in (D). (E) Discontinuity frequencies of lymphatics in control *vs.* post-RL and example in (F). CRLM control (n = 253 lymphatics; n = 3 patients), CRLM post-RL (n = 426 lymphatics; n = 4 patients), HCC control (n = 413 lymphatics; n = 3 patients) and HCC post-RL (n = 340 lymphatics; n = 5 patients). Scale bars indicate 50 μm ∗*p* <0.05, ∗∗*p* <0.01, two-tailed Student’s *t* test. CRLM, colorectal liver metastases; HCC, hepatocellular carcinoma; RL, radiation lobectomy.

We scored lymph vessels (n = 1,432) on aberrant morphology, including vessel wall tortuosity, thickness and discontinuity.[13], [24], [25], [26] There were no statistically significant differences in lymph vessel morphology between non-radioembolized CRLM and non-radioembolized HCC tumors (*p* >0.05 for all morphological characteristics). However, post-RL lymph vessels, when compared to naïve or chemotherapy-treated vessels, were more often tortuous (Fig. 2A,B and Table 3, *p* <0.01 for CRLM samples, *p* <0.05 for HCC). Post-RL lymph vessel walls were also thickened (Fig. 2C,D*, p* <0.05 in both CRLM and HCC samples), and frequently displayed discontinuous, misaligned vessel walls (Fig. 2E,F*, p* <0.05 in both groups).

**Table 3:** Morphological characteristics of lymph vessels.

	CRLM			HCC		
	Control (n = 3)	RL (n = 7)	*p* value§	Control (n = 3)	RL (n = 6)	*p* value§
Tortuosity (%)	7.0 ± 3.0	35.0 ± 5.8	<0.01	12.4 ± 5.8	46.9 ± 8.2	<0.05
Thickened (%)	3.9 ± 1.5	32.1 ± 6.8	<0.05	6.9 ± 4.5	28.4 ± 4.8	<0.05
Discontinuous (%)	4.2 ± 1.0	15.9 ± 2.8	<0.05	3.8 ± 1.0	13.4 ± 2.7	<0.05
Vessel density (vessels/mm^2^)	4.9 ± 1.5	10.6 ± 2.4	n.s.	16.8 ± 6.9	14.2 ± 9	n.s.
Luminal area (μm^2^)	283 ± 16.9	433 ± 19.5	<0.0001	290 ± 16.9	503 ± 29.3	<0.0001

CRLM, colorectal liver metastases; HCC, hepatocellular carcinoma; RL, radiation lobectomy.

Numbers as mean ± SEM. Student's *t* test was used for tortuosity, thickness and discontinuity. Mann-Whitney *U* test was used for vessel density and luminal area.

§ Comparing non-radioembolized *vs*. radioembolized vessels.

**Fig. 3:**
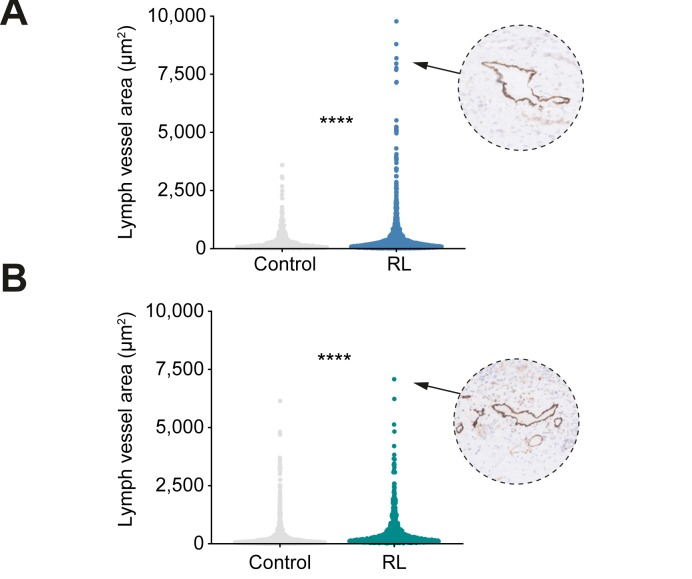
Lymphatics have higher luminal size post-RL. (A). Lumen area per lymph vessel for controls with CRLM (average of 283 μm^2^, n = 569 lymphatics; n = 3 patients), and after RL (433 μm^2^, n = 1,773 lymphatics; n = 7 patients). (B). Per vessel lumen area in controls with HCC (290 μm^2^, n = 999 lymphatics; n = 3 patients) and after RL (503 μm^2^, *n* = 654 lymphatics; *n* = 6 patients). Insets indicate examples of large lymphatic vessels. ∗∗∗∗*p* <10^-4^, Mann-Whitney *U* test. CRLM, colorectal liver metastases; HCC, hepatocellular carcinoma; RL, radiation lobectomy.

Dysfunctional lymph vessels cannot drain interstitial fluid, leading to stasis within the lymph vessels and thus resulting in large lumina.^27^^,^^28^ Analysis of 5,695 manually annotated peritumoral lymph vessels revealed that peritumor lymph vessels of non-radioembolized CRLM and non-radioembolized HCC had similar luminal size (283 ± 16.9 μm^2^ in CRLM *vs.* 290 ± 16.9 μm^2^ in HCC, *p* >0.05). After radioembolization, a 1.5-fold increase in luminal size in CRLM samples was noted (*p* <0.0001, Fig. 3A and Table 3). A similar 1.7-fold increase in irradiated HCC vessels was observed (*p* <0.0001, Fig. 2B and Table 3). We frequently found the presence of vessels that were more than 25-fold larger than the median, especially in the CRLM group (Fig. 3A,B). Consistent with previous reports,^29^^,^^30^ peritumoral lymph vessel density of non-radioembolized HCC tumors was higher than that of non-radioembolized CRLM tumors, albeit not statistically significant (4.9 ± 1.5 vessels/mm^2^ in CRLM *vs.* 16.8 ± 6.9 vessels/mm^2^ in HCC, *p* >0.05). We found no difference in the lymph vessel density between non-irradiated and irradiated samples (*p* = n.s. in both tumor groups).

### Absence of lymphatic drainage in radioembolized livers

**Fig. 4:**
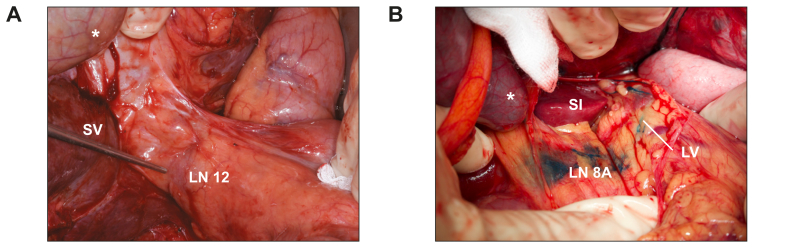
Intraoperative liver lymphangiography reveals absent lymph drainage in post-RL patients. (A). After injection of patent blue dye in the parenchyma of a previously radioembolized liver, no blue staining is observed in perihilar lymph nodes or LV (depicted here is LN station 12). (B). Intraoperative liver lymphangiography in a non-irradiated control patient shows lymph vessels draining blue-dyed fluid towards LN 8A. ∗Gallbladder. LN, lymph node; LV, lymph vessel; RL, radiation lobectomy; S, segment.

Next, we performed ILL in eight post-RL patients (n = 4 CRLM, n = 4 HCC). Only one patient (1/8) had blue staining of lymph node station 12 left to the hepatoduodenal ligament and another patient (1/8) had staining of a small lymph vessel right to the hepatoduodenal ligament (Fig. 4A, Table S1). We compared these patients to a cohort previously published by our group of ten patients (n = 9 CRLM, n = 1 HCC) and two additional non-radioembolized patients with HCC. In this control group, all 12 patients exhibited clear blue staining of the ipsilateral hepatic pedicle lymph nodes (12/12) and lymph vessels (12/12) upon patent blue injection (Fig. 4B, *p* = 1.0x10^-4^, Fisher’s exact test when compared to RL group). While these results imply radioembolization is associated with decreased lymphatic drainage, pathophysiological characteristics of the liver, such as fibrosis/cirrhosis,[31], [32], [33], [34] steatosis,^35^ and hepatitis,^36^^,^^37^ have previously been reported to influence lymph vessel function. Because the small sample size precluded formal multivariate (logistic) regression analysis to account for such confounders,^38^ we evaluated whether these characteristics were statistically different between radioembolized (cases) and non-radioembolized (control) patients. We found no difference between cases and controls in the prevalence of steatosis (*p* = 0.37, Fisher’s exact test), hepatitis, fibrosis or cirrhosis (all *p* = 1). Moreover, there were no statistically significant difference between the groups in terms of tumor type (HCC or CRLM, *p* = 0.39), major or minor surgery (*p* = 0.17), sex (*p* = 0.62), age (*p* = 0.9) or preoperative chemotherapy status (*p* = 1) (Table 2 includes all potential confounders that were tested). Thus, these results suggest that radioembolization reduces the drainage of lymph from intrahepatic lymph vessels into perihilar lymph nodes and vessels.

## Discussion

This study assessed lymph vessel morphology and function in post-RL livers through IHC analysis of resection specimens and ILL. Irradiated lymphatics displayed features of aberrant morphology, including tortuous, thick, and irregularly shaped vessel walls. Moreover, post-RL lymphatic vessels were found to be dysfunctional, as was indicated by their dilated lumens, suggesting lymph stasis, and the absence of lymph drainage to perihilar lymph nodes and lymph vessels.

Although this study is the first to analyze the effects of radioembolization on liver lymph vessels, radiation-induced lymph edema is a well-known side effect following radiation therapy for breast cancer.^39^ In a study comparing non-irradiated and irradiated melanoma samples, radiation resulted in enlarged cutaneous lymph vessels with abnormal morphology within 4 weeks, and increased vessel density through lymphangiogenesis within 1 year.^40^ Moreover, it was found that radiation induces lymphatic endothelial cell apoptosis and lymphatic dysfunction through fibrosis.^13^ Essentially, lymph vessels in post-RL livers resemble those seen in transgenic mouse models of defective lymph angiogenesis.^24^^,^^25^ The impaired lymph drainage in the current study may thus have a dual etiology: (i) radiation-induced fibrosis, leading to blockage of lymph flow and subsequently ectatic vessels through lymph stasis, and (ii) abnormal lymph vessel morphology, either through radiation-induced apoptosis, or uncoordinated lymph angiogenesis, or both. The perihepatic lymph nodes are unlikely to block the lymph drainage: although lymph nodes may become fibrotic upon radiation damage,^26^ the mean tissue penetration of the microsphere’s beta emission is 2.5 mm. Hence, it may be assumed that all energy is absorbed by the liver lymph vessels within the parenchyma.

Lymphatic vessels are a frequent route for cancer dissemination, and extrahepatic metastatic spread to lymph nodes is a major negative determinant of outcome in both primary and secondary liver cancers.[14], [15], [16] Experimental work has shown that VEGF-C expressed by tumor cells increases lymphatic metastasis through several pathophysiological mechanisms including the induction of peritumor lymphatic hyperplasia, and the expression of VEGF-C has been associated with recurrence in lymph nodes and distant sites for both HCC^41^ and CRLM.^16^ Therefore, targeting lymph vessels represents an interesting therapeutic opportunity.^18^ Moreover, numerous drugs currently in clinical trials inhibit lymphatic metastasis-promoting molecular pathways, thus underscoring the potential importance of strategies to block the dissemination of cancer cells via the lymphatics.^18^^,^^42^

From a surgical point of view, these findings underscore the rationale for using radioembolization (RL) rather than portal venous embolization as neoadjuvant therapy in the context of insufficient future liver remnant. During portal venous embolization, the portal vein of the ipsilateral, tumor-bearing liver lobe is embolized.^43^ As a result, portal blood shunts towards the contralateral liver lobe and induces its growth, enabling safe resection. Portal vein embolization is associated with a low complication rate and results in future liver remnant hypertrophy within 4-6 weeks.^43^ However, portal vein embolization does not treat the tumor, which is disadvantageous in patients with HCC for whom effective neoadjuvant therapy is lacking. A number of studies have demonstrated accelerated progression of existing tumors after portal vein embolization.[44], [45], [46] Conversely, approximately 30% to 50% of patients with CRLM or HCC, respectively, have a complete to partial response following radioembolization.^47^^,^^48^ In a meta-analysis, patients with HCC treated with ^90^Y radioembolization had better progression free-survival than those treated with transarterial chemoembolization.^49^ We and others have recently demonstated that major hepatectomy following RL can be safely performed.^7^ In light of these and the present results, we speculate that RL could control tumor growth and further spread while waiting for future liver remnant hypertrophy.

Limitations of this study include the small sample sizes. Moreover, despite the use of digital pathology software, immunohistochemical analyses remain semi-quantitative. Complete blinding was not possible due to the presence of microspheres in post-RL specimens. Finally, it was not possible to correlate lymphatic vessel dysfunction with lymphatic vessel invasion due to the scarcity of the latter event. Future studies in larger samples could reveal such events, as well as whether radioembolization is associated with reduced progression to lymph drainage-specific sites such as the perihepatic lymph nodes and lungs.

In conclusion, this study demonstrates that radioembolization impairs the function of peritumoral lymph vessels. Whether this impaired function may attenuate metastatic spread via this route warrants further translational and clinical research.

## Financial support

This research was supported by a private fund.

## Authors’ contributions

DA designed the analysis, collected and contributed data and performed the analysis; LvdB designed the analysis, collected and contributed data; MML, MGEHL, MLJS, IQM, JdB contributed data; OK conceived and designed the analysis; IHMBR and JH conceived and designed the analysis, collected and contributed data. All authors contributed to the writing of the paper.

## Data availability statement

The data supporting the findings of this study are partly available within the supplementary materials and at 10.1093/bjs/znac076. All other data are available from the corresponding author upon reasonable request.

## Conflict of interest

Marnix Lam is a consultant for Boston Scientific and Terumo. Maarten Smits is consultant for Philips and Terumo/Quierem Medical and has served as speakers for SirTex, BTG, Swedisch Orphan Biovitrum, Terumo and Metronic. The Department of Radiology and Nuclear Medicine of the UMC Utrecht receives royalties from Quirem Medical. No other potential conflict of interest relevant to this article was reported.

Please refer to the accompanying ICMJE disclosure forms for further details.
