# Supplementary material for: 90Y-/166Ho- ‘Radiation lobectomy’ for liver tumors induces abnormal morphology and impaired drainage of peritumor lymphatics
Source: JHEP Rep. 2023 Dec 5;6(2):100981. doi: 10.1016/j.jhepr.2023.100981 (PMC10827593; doi:10.1016/j.jhepr.2023.100981)
Supplement: Multimedia component 1 [file mmc1.pdf]

^{90}Y-/^{166}Ho- ‘Radiation lobectomy’ for liver tumors induces abnormal morphology and impaired drainage of peritumor lymphatics

Daan Andel, Lotte van den Bent, Marnix Gerard Ernest Hendrik Lam, Maarten Leonard Johannes Smits, Isaac Quintus Molenaar, Joep de Bruijne, Miangela Marie Lacle, Onno Kranenburg, Inne Hildbrand Max Borel Rinkes, Jeroen Hagendoorn

Table of contents

Supplementary data file 1……………………………………………..…………..………2

Fig. S1……………………………………………………………………………………5

Fig. S2……………………………………………………………………………………6

Table S1………………………………………………………………………………… 7

Supplementary reference…………………………………………………………..…….. 8

Supplementary data file 1

LYMPHANGIOGRAFIE / LIVER RESECTION – CRF

Hospital: ………..
Patient number: ………..
OK: date: ………..

Surgeon:

1th assistent:

Indication (tumor type):
Primary tumor in situ (CRLM):
Preop. Chemo:
Preop. Y90:
Procedure performed (wig/segments etc.):
Open / robot:
Injection Patent Blue in segment:
Total ml injected (number of fractions):
Lymph vessels on capsule blue (accidental): yes/no
Time injection untill blue staining of lymph vessels in ligament or nodes (minutes):
Video or photos procedure:

Nodes

**Yes* No-**	**Lymph station**	**After how many minutes.***	**Description**	**PA vial #**
-	12right		Right side ligament (along bile duct/porta)	
-	12left		Left side ligament (along a hep sinistra)	
-	13		Head of pancrease/base of ligament	
-	8A		A hep communis	
-	9		Truncus	
-	7		Omentus minus	
	~		D. cysticus	

-	~		Suprahepatische VCI (left/right)	
	~		Other, nl.:	

***** Total minutes between injection and first blue staining of node***

Lymph vessels visible (blue discoloration)

Yes* No-	Location
-	Right side ligament
-	Left side ligament
-	In parenchyma during transection
-	Along right / middle / left hepatic vein
-	Diaphragm
-	Omentum minus

Arterial anatomy

Normal yes/no

If anatomical variant, describe:

Replaced/accessory AHD from the AMS

Replaced/accessory a. hep. sinistra from the gastrica sinistra

Other, namely………

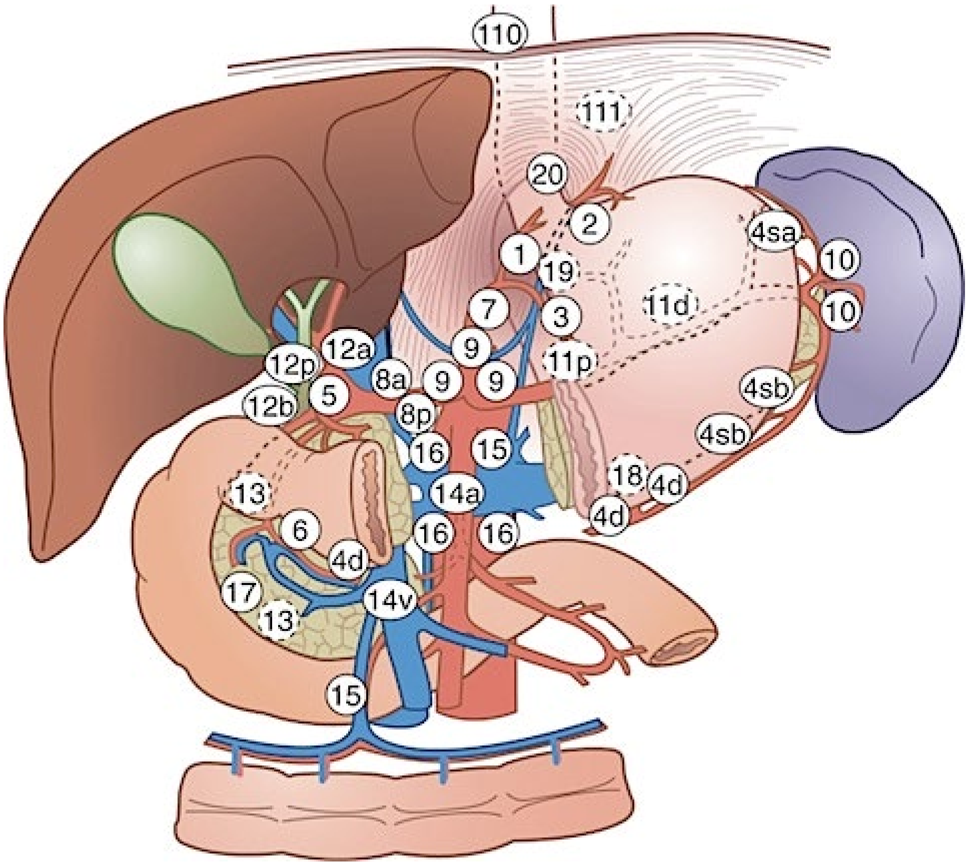
110
111
20
2
4sa
10
1
19
10
7
3
11d
9
12a
11p
12p
8a
9
9
4sb
5
12b
8p
16
15
4sb
13
14a
18
4d
4d
6
16
16
4d
17
14v
13
15

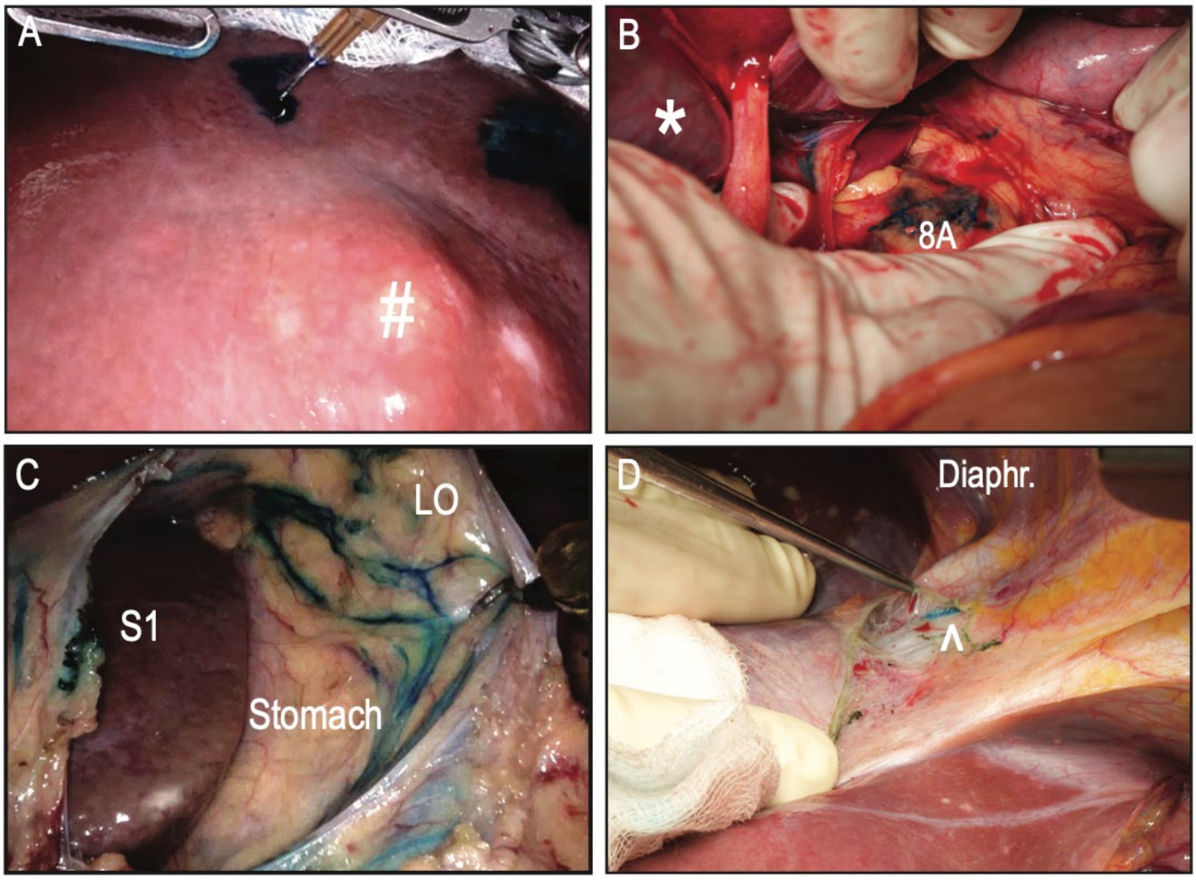

Fig. S1. Procedures of intraoperative liver lymphangiography
(A). Injection if Patent blue dye 2 cm away from the tumor (#). (B). After injection of dye, the perihilar region is inspected for blue staining. Here, lymph node station 8A is depicted. (C). Visualisation of the lesser omentum, containing blue stained lymphatic vessels. (D). Blue-stained lymphatic vessels (arrow) are seen along the suprahepatic inferior vena cava. Note that the figures are not from the same procedure. The procedures were performed in patients with non-radioembolized livers.

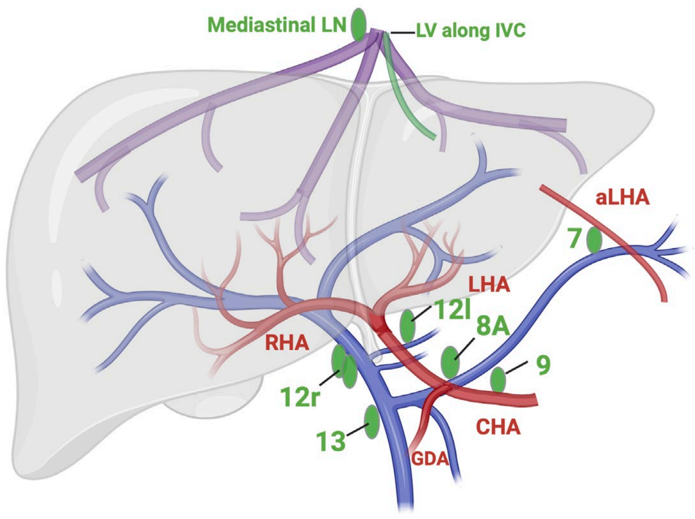

Fig. S2. Schematic representation of perihepatic lymph nodes. Green circles represent lymph nodes. Blue and purple vessels represent portal and hepatic veins, respectively. Red vessels represent hepatic arteries. aLHA: accessory/replaced left hepatic artery, CHA: common hepatic artery, GDA: gastroduodenal artery, IVC: inferior vena cava, LHA: left hepatic artery, LV: lymph vessel, RHA: right hepatic artery.

Table S1. Intraoperative liver lymphangiography details

	ILL1	ILL2	ILL3	ILL4	ILL5	ILL6	ILL7	ILL8	ILL9	ILL10
**Tumour type**	CRLM	CRLM	CRLM	CRLM	HCC	HCC	HCC	HCC	HCC	HCC
**Case/control**	Case	Case	Case	Case	Case	Case	Case	Case	Control	Control
**Injection Patent blue**										
Location	S5	S6/S7	S7	S2/S3	S6	S7	S8	S6	S6/7	S4A
Volume (mL)	2	4	3	3	3	2	2	3	4	4
Time to staining (min)	5	NA	NA	2	NA	NA	NA	NA	6	
**LN station / location**										
12 right	-	-	-	-	-	-	-	-	-	X
12 left	-	-	-	X	-	-	-	-	-	X
13	-	-	-	-	-	-	-	-	-	
8A	-	-	-	-	-	-	-	-	X	X
9	-	-	-	-	-	-	-	-	-	-
7	-	-	-	-	-	-	-	-	-	-
D. cysticus	-	-	-	-	-	-	-	-	-	-
Suprahepatic VCI right	-	-	-	-	-	-	-	-	-	-
Suprahepatic VCI left	-	-	-	-	-	-	-	-	-	-
Other	-	-	-	-	-	-	-	-	-	-
**LV location**										
Hepatoduodenal lig. Right	X	-	-	-	-	-	-	-	X	-
Hepatoduodenal lig. Left	-	-	-	-	-	-	-	-	X	X
Parenchymal	-	-	-	-	-	-	-	-	-	-
Left hepatic vein	-	-	-	-	-	-	-	-	-	-
Middle hepatic vein	-	-	-	-	-	-	-	-	-	-
Right hepatic vein	-	-	-	-	-	-	-	-	-	-
Diaphragm	-	-	-	-	-	-	-	-	-	-
Omentum minus	-	-	-	-	-	-	-	-	-	-
Other	-	-	-	-	-	-	-	-	-	-

'X' indicates blue staining was observed, CRLM: colorectal liver metastases, HCC: hepatocellular carcinoma, LN: lymph node, LV: lymph vessel. Only those patients that were assessed in this study were included. All other (control) patients are described in *'van den Bent et al., BJS, 2022'* (1).

Supplementary reference

1. van den Bent L, Frenkel NC, Poghosyan S, Molenaar IQ, Padera TP, Kranenburg O, et al. Liver lymphatic drainage patterns follow segmental anatomy. British Journal of Surgery. 2022 May 16;109(6):559–60.
