# Supplementary material for: 90Y-/166Ho- ‘Radiation lobectomy’ for liver tumors induces abnormal morphology and impaired drainage of peritumor lymphatics
Source: JHEP Rep. 2023 Dec 5;6(2):100981. doi: 10.1016/j.jhepr.2023.100981 (PMC10827593; doi:10.1016/j.jhepr.2023.100981)
Supplement: Multimedia component 4 [file mmc4.pdf]

Research article

JHEP|Reports

^{90}Y-/^{166}Ho- 'Radiation lobectomy' for liver tumors induces abnormal morphology and impaired drainage of peritumor lymphatics

Daan Andel,[1,2,*] Lotte van den Bent,[1,2] Marnix Gerard Ernest Hendrik Lam,[3] Maarten Leonard Johannes Smits,[3] Isaac Quintus Molenaar,[1] Joep de Bruijne,[4] Miangela Marie Laclé,[5] Onno Kranenburg,[1,2] Inne Hildbrand Max Borel Rinkes,[1,2] Jeroen Hagendoorn[1,2,*]

[1]Department of Surgical Oncology, University Medical Center Utrecht, Cancer Center, Utrecht, The Netherlands; [2]Laboratory for Translational Oncology, University Medical Center Utrecht, Cancer Center, Utrecht, The Netherlands; [3]Department of Radiology and Nuclear Medicine, University Medical Center Utrecht, Cancer Center, Utrecht, The Netherlands; [4]Department Gastroenterology and Hepatology, University Medical Center Utrecht, Utrecht, the Netherlands; [5]Department of Pathology, University Medical Center Utrecht, Utrecht University, Utrecht, The Netherlands

JHEP Reports **2024.** https://doi.org/10.1016/j.jhepr.2023.100981

Background & Aims: High-dose unilobar radioembolization, or 'radiation lobectomy' (RL), is an induction therapy that achieves contralateral future liver remnant hypertrophy while simultaneously irradiating the tumor. As such, it may prevent further growth, but it is unknown whether RL affects intrahepatic lymphatics, a major route via which liver tumors disseminate.
Methods: This was a case-control study conducted at University Medical Center Utrecht. The study compared lymph vessels in livers that had undergone RL (cases) with those in livers that had not undergone RL (controls). Histological samples were acquired from patients diagnosed with hepatocellular carcinoma (HCC) or colorectal liver metastases (CRLM) between 2017 and 2022. Lymph vessel morphology was analyzed by two researchers using podoplanin, a protein that is expressed in lymphatic endothelium. *In vivo* liver lymph drainage of radioembolized livers was assessed using intraoperative liver lymphangiography (ILL): during liver surgery, patent blue dye was injected into the liver parenchyma, followed by inspection for staining of perihepatic lymph structures. ILL results were compared to a previously published cohort.
Results: Immunohistochemical analysis on post-RL tumor tissues from ten patients with CRLM and nine patients with HCC revealed aberrant morphology of irradiated liver lymphatics when compared to controls (n = 3 per group). Irradiated lymphatics were tortuous (p <0.05), thickened (p <0.05) and discontinuous (p <0.05). Moreover, post-RL lymphatics had larger lumens (1.5–1.7x, p <0.0001), indicating lymph stasis. ILL revealed diminished lymphatic drainage to perihepatic lymph nodes and vessels in irradiated livers when compared to non-radioembolized controls ($p = 1.0x10^{-4}$).
Conclusions: Radioembolization impairs peritumoral lymph vessel function. Further research is needed to evaluate if radioembolization impairs tumor dissemination via this route.
Impact and implications: Unilobar radioembolization can serve as an alternative to portal venous embolization for patients who are considered unresectable due to an insufficient future liver remnant. This research suggests that radioembolization impairs the function of peritumoral liver lymph vessels, potentially hindering dissemination via this route. These findings provide support for considering unilobar radioembolization over standard portal venous embolization.
© 2023 The Authors. Published by Elsevier B.V. on behalf of European Association for the Study of the Liver (EASL). This is an open access article under the CC BY license (http://creativecommons.org/licenses/by/4.0/).

Introduction

Radioembolization can be an effective treatment for primary and metastatic liver cancers.[1,2] It uses the predominantly arterial blood supply of liver tumors to locally administrate b-radiation-emitting microspheres. For hepatocellular carcinoma (HCC), radioembolization has recently been added to the BCLC strategy for very early-to intermediate stage disease in patients who are not candidates for thermal ablation or resection.[3] For colorectal liver metastases (CRLM), radioembolization is an option for patients with unresectable, refractory and liver-dominant disease.[2] In both HCC and CRLM, radioembolization can be used as neoadjuvant treatment for patients that are deemed unresectable due to insufficiency of the future liver remnant.[4–7] In these cases, high-dose, unilobar treatment may induce ipsilateral atrophy (thus termed 'radiation lobectomy' [RL]) and contralateral hypertrophy of the future liver remnant, while simultaneously treating the tumor.[5]

Keywords: Lymphatics; Radiation lobectomy; Radioembolization; Intraoperative liver lymphangiography; hcc; crlm; Immunohistochemistry.
Received 7 July 2023; received in revised form 26 October 2023; accepted 21 November 2023; available online 5 December 2023
* Corresponding authors. Address: PO BOX 85500, 3508 GA, Utrecht, The Netherlands; Tel.: +31 (0)88 75 598 11 (D. Andel), or +31 (0)88 75 569 6831 (J. Hagendoorn).
E-mail addresses: D.S.H.Andel-3@umcutrecht.nl (D. Andel), j.hagendoorn-3@umcutrecht.nl (J. Hagendoorn).

ELSEVIER

EASL The Home of Hepatology

The main antitumor effects of radiation are attributed to DNA damage – either directly or through the formation of oxygen radicals,[8] as well as the activity of the tumor microenvironment. Important determinants include activity of cancer-associated fibroblasts,[9] modulation of the extracellular matrix,[10] antitumor immunity[8,11] as well as endothelial cell death and subsequent hypoxia.[12] In addition, radiation causes apoptosis of lymphatic endothelial cells, loss of function of lymphatic vessels and subsequent lymphedema.[13] All of these factors can influence tumor response, progression and dissemination through blood vessels and/or lymph vessels.

The pathophysiological importance of the liver lymphatic system is underscored by the large flux of liver lymph to the thoracic duct, which allows cells to spread to the lungs, and the frequent presence of metastases in hepatic pedicle lymph nodes.[14,15] Moreover, the prognosis of patients with liver malignancies and metastases to the lymph nodes is worse than in those without lymph node involvement.[14–16] As endothelial cells are vulnerable to irradiation, the loss of lymphatic function induced by radioembolization could potentially hinder the dissemination of cancer cells through these routes.[16–18] Our group recently conducted two studies using liver lymphangiography, which demonstrated that lymph drainage follows the segmental anatomy of the liver.[19,20] However, lymphatic function has not been characterized in radioembolized livers. In this study, we hypothesized that irradiated liver lymph vessels become dysfunctional. To evaluate this hypothesis, we analyzed lymphatic morphology in post-radioembolization tumor tissues and assessed lymph vessel function through intraoperative liver lymphangiography.

Patients and methods

Study setup and patient selection

This was a case-control study conducted at University Medical Center (UMC) Utrecht. The study aimed to compare lymph vessels in livers that had undergone RL (cases) with those in livers that had not undergone RL (controls). This comparison was conducted using two methods: (i) immunohistochemical (IHC) analysis to assess lymph vessel morphology and (ii) intraoperative liver lymphangiography (ILL) to evaluate liver lymphatic drainage to perihilar lymph nodes and lymph vessels.

For the IHC analyses, histological sections were obtained from patients with HCC or CRLM who received RL and hemihepatectomy between 2017 and 2022. Controls consisted of patients who underwent hemihepatectomy without prior RL and were selected from the surgical pathology files. Cases and controls were matched for tumor type, extent and size, age, preoperative chemotherapy status and metastatic lymph node involvement.

For the ILL analyses (refer to the 'Intraoperative liver lymphangiography' section for procedure details), we utilized the established framework from the LILY study. The LILY study's objective was to map the drainage patterns of the liver lymphatic system using patent blue dye in patients without a history of prior liver surgery or liver irradiation.[19] ILL is routinely used during liver resection at UMC Utrecht to assess (potentially) tumor-draining lymph nodes. For the case group, we mapped draining patterns in all patients with CRLM or HCC who had undergone prior RL between 2022 and 2023 and for whom intraoperative liver lymphangiography was clinically indicated. Patients with a prior history of liver radiation, other than radioembolization, or prior liver surgery were not included in the study as cases. As controls, we reanalyzed the previously published data from the LILY cohort, focusing on patients with HCC or CRLM. Additionally, we mapped lymph drainage in two non-radioembolized patients with HCC. We extracted confounding factors that could potentially influence lymphatic drainage, such as cirrhosis, fibrosis, hepatitis, and steatosis from the patients' electronic health records and pathology files.

The need for written informed consent was waived by the UMC Utrecht Institutional Review Board (reference 21/625, 10.12.22). The study was performed in accordance with the Declaration of Helsinki.

Radiation lobectomy

All patients underwent unilobar radioembolization with the intent of inducing concomitant ipsilateral atrophy and contralateral (future liver remnant) hypertrophy (RL setting). The procedure was performed by a board-certified interventional radiologist (MLJS) and nuclear physician (MGEHL). Patients had to be chemotherapy naïve for at least 4 weeks prior to RL. Patients underwent a work-up angiography for safety and treatment planning. During this angiography, the hepatic arterial anatomy was mapped, sources of extrahepatic shunting were identified and addressed, and a scout dose was administered at selected injection position(s). C-arm CT with contrast injection through the intra-arterial catheter was typically performed at the injection position of each scout dose. The scout dose consisted of technetium-99m macroalbumin aggregates (^{99m}Tc-MAA) in patients treated with yttrium-90 (^{90}Y) microspheres (Therasphere®, Boston Scientific) or holmium-166 (^{166}Ho) scout (QuiremScout®, Terumo) in patients treated with ^{166}Ho microspheres (QuiremSpheres®, Terumo). Work-up angiography was followed by SPECT-CT (single photon emission computed tomography) to determine the distribution of the scout dose. This information was used for safety (no excessive radiation to non-target structures) and for calculating the prescribed activity. The prescribed activity was calculated using the medical internal radiation dose mono-compartment method for ^{90}Y glass and ^{166}Ho microspheres, or the partition model in a selection of ^{166}Ho-treated patients. The desired doses in the medical internal radiation dose model (MIRD) were 80–120 Gy for ^{90}Y and 60 Gy for ^{166}Ho.[21] The actual RL treatment was performed no later than 2 weeks following the scout dose. The treatment dose was administered under angiography similar to the work-up angiography. All patients received unilobar treatment with or without segmentectomy. Segmentectomy was performed by administering microspheres into one of the segmental arteries. The dose depended on microsphere activity and tumor volume, but it typically exceeded 200 Gy. After RL, patients received follow-up imaging consisting of ^{99m}Tc-mebrofenin hepatobiliary scintigraphy and CT or MRI scans at a 3-monthly interval to assess treatment effect and resectability.

Surgery

The decision to resect was made after discussion in an hepato-pancreato-biliary (HPB) multidisciplinary tumor board. Patients were deemed candidates for resection in case of stable oncological disease. The future liver remnant was deemed sufficient for surgery if the function exceeded 2.69 %/min/m^2 on hepatobiliary scintigraphy.[22] The extent of the resection was

primarily based on the segments embolized and the function of the liver remnant.

Intraoperative liver lymphangiography

During the liver resection, ILL was performed by three board-certified HPB surgeons (IQM, JH, and IHMBR). Please refer to Fig. 1 (schematic) and Fig. S1 for a summary of the various steps involved in the lymphangiography procedure using patent blue dye. Patent blue dye is a high molecular weight protein tracer that is selectively absorbed by lymphatic vessels (Fig. 1). It is commonly used in clinical practice for lymph node mapping.

After entering the abdominal wall and visualizing the liver and tumor regions, 2 to 4 mL of patent blue dye were injected in 2 to 4 fractions (Fig. S1A). The dye was injected deep into the liver parenchyma, within two centimeters of the tumor, using a 15/20G needle. Subsequently, the liver was mobilized, and a waiting period of 5 to 10 min was allowed to permit the tracer to be absorbed by lymphatic vessels and transported to extraparenchymal lymph nodes and vessels. The examination for blue discoloration involved systematically inspecting perihepatic lymph vessels and lymph nodes, starting from the liver hilum and progressing towards the duodenum along the hepatoduodenal ligament (Fig. S1B). Following this, the minor omentum/gastrohepatic ligament was inspected (Fig. S1C), followed by the examination of lymph nodes along the suprahepatic inferior vena cava (Fig. S1D). This assessment included lymph nodes at stations 12r and 13 on the right side of the hepatoduodenal ligament, 12l on the left side of the hepatoduodenal ligament, 8A and 9 along the common hepatic artery, and station 7 in the minor omentum/gastrohepatic ligament (Fig. S2). Mediastinal nodes were inaccessible for direct observation.

An HPB surgeon filled out a case report form to document the injection site, the total volume of dye used, the time until the first staining of perihepatic lymph nodes or lymph vessels, and the exact anatomical location of stained lymph nodes and vessels (please see supplementary data file 1 for the case report form). Digital images were captured whenever feasible, and lymph nodes were excised as deemed appropriate by the surgeon.

Immunohistochemistry

Stainings were performed on 4 μm thick formalin-fixed paraffin embedded sections mounted on a glass microscope slide. Formalin-fixed paraffin embedded sections were dewaxed and rehydrated, after which endogenous peroxidase was blocked with 1.5% hydrogen peroxide in phosphate-buffered saline. Heat-mediated antigen retrieval was performed in citrate buffer. Slides were incubated with a primary antibody for the lymphatic endothelial cell marker podoplanin (Sigma-Aldrich; HPA007534) overnight at 4 °C. The secondary antibody was incubated for 1 h at room temperature. 3,3′-diaminobenzidine was used as a chromogen and hematoxylin was used as a counterstain.

Qualitative and quantitative analyses

Analyses of IHC data were performed by DA and LvdB, under the guidance of a board-certified pathologist (MML). Slides were digitized and peritumor regions were manually annotated using QuPath version 0.2.3. 'Peritumor' was defined as a 1 mm-thick region surrounding the tumor. Regions with extensive stroma were excluded because of false positive background staining of fibroblasts. For the qualitative analyses, a high-density peritumor region of 10 mm^2 was selected for each tumor, wherein the percentage of tortuous, discontinuous, and thick-walled lymphatic vessels was calculated. Tortuosity was defined as the ratio of the length of the vessel wall to the longest diameter of the lymphatic vessel, corrected for the area of the lumen.[23] For the quantitative analysis, each lymphatic vessel within the peritumor region was manually annotated, including the vessel lumen, to allow extraction of per vessel characteristics. Tumors in which no region could be found with at least 30 blood vessels were excluded (n = 4). Blinding was not possible in this study because of the frequent presence of microspheres in the pathology slides.

Statistical analyses

Descriptive statistics are denoted as means ± standard deviation or medians (range) for age. Statistical analysis was performed in R version 4.2.0. Means (± standard error of the mean) were compared using the unpaired, two-tailed Student's *t* test or

Table 1. Patient characteristics of the IHC cohort.

	IHC cohort (n = 19)			
	CRLM		HCC	
	Control (n = 3)	RL (n = 7)	Control (n = 3)	RL (n = 6)
Demographics				
Sex (M/F)	2/1	5/2	2/1	6/0
Age (years)	76 (64–83)	58 (46–78)	71 (71–76)	75 (63–79)
Baseline				
Cirrhosis	0	0	1	1
Previous chemo	2	7	0	0
Treatment details				
Microsphere (^{90}Y/^{166}Ho)	NA	5/2	n.a.	6/0
Time treatment to surgery (days)*	23 and 61	118 ± 39	n.a.	167 ± 62
Surgery				
Major/minor**	0/3	7/0	1/2	6/0

^{166}Ho, holmium-166; ^{90}Y, yttrium-90; CRLM, colorectal liver metastases; HCC, hepatocellular carcinoma; IHC, immunohistochemistry; RL, radiation lobectomy.
Demographics, baseline characteristics and treatment details of the immunohistochemistry cohort. Data are numbers, median (range) or mean ± standard deviation. Age at the time of liver surgery.
* The time from treatment to surgery indicates the time between the last treatment (RL in the case group, systemic treatment in the CRLM control group) and hepatectomy.
** Major: (extended) hemihepatectomy; minor: wedge or segment resection(s).

Table 2. Patient characteristics of the ILL cohort.

	ILL (n = 20)		
	**Control[1] (n = 12)**	**RL (n = 8)**	***p* value[§]**
Demographics			
Sex (M/F)	7/5	6/2	0.64
Tumor (CRLM/HCC)	9/3	4/4	0.17
Age (years)	66 [50-80]	67 [48-76]	0.91
Baseline characteristics			
Cirrhosis	1	0	1
Fibrosis	4	3	1
Hepatitis	3	3	1
Steatosis	4	5	0.37
Previous treatment			
Chemotherapy	5	4	1
PVE	4	1	0.60
Treatment details			
Microsphere ($^{90}Y/^{166}Ho$)	n.a.	3/5	n.a.
Time treatment to surgery (days)*	56 ± 52	97 ± 25	0.17
Surgery			
Major/minor**	8/4	8/0	0.12

^{166}Ho, holmium-166, ^{90}Y, yttrium-90; CRLM, colorectal liver metastases; HCC, hepatocellular carcinoma; ILL, intraoperative liver lymphangiography; PVE, portal vein embolization; RL, radiation lobectomy.
Demographics, baseline characteristics and treatment details. Age at time of surgery as median (range).
[1] In part obtained from 'van den Bent *et al.*, BJS, 2022'.
* The time from treatment to surgery indicates the time between the last treatment (RL in the case group, systemic treatment in the control group, if received) and hepatectomy.
** Major: (extended) hemihepatectomy; minor: wedge or segment resection(s).
§ Difference between radioembolized (RL) and non-radioembolized (Control) group. Fisher's exact test and Mann-Whitney *U* test for categorical and numerical variables, respectively.

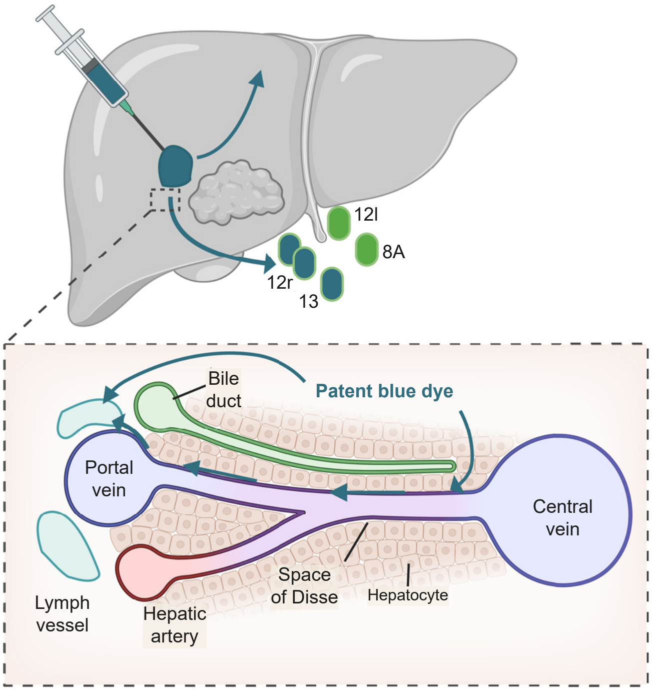

Fig. 1. Schematic depicting the injection of patent blue dye and intrahepatic lymph flow. After injection, patent blue dye flows directly or via the Space of Disse into intrahepatic lymph vessels. The dye is then drained from these lymph vessels into extrahepatic lymph nodes and vessels located near the hepatoduodenal ligament, lesser omentum, and suprahepatic area toward the inferior vena cava. It is important to note that lymph vessels follow a segmental anatomy; for instance, the injection of patent blue dye into the right lobe will result in blue staining of nodes on the right side of the ligament, such as lymph stations 12r and 13 [19,20].

Mann-Whitney *U* test, where appropriate. To compare lymphatic drainage between radioembolized and non-radioembolized patients, we used the Fisher's exact test to determine if there was a significant association between previous RL and the presence or absence of patent blue dye staining in perihepatic lymph nodes or vessels.

Results

Patient characteristics

The IHC cohort (n = 19 total) included post-hepatectomy tumor tissues from three non-irradiated controls with CRLM and seven patients with CRLM who underwent RL (cases). Moreover, this cohort included three controls with HCC and six patients with HCC who underwent RL. Table 1 displays the patient demographics, baseline characteristics, and treatment details of the IHC cohort. In the CRLM group, two controls and seven cases received preoperative chemotherapy, while in the HCC group, all patients were chemonaive. Two patients with HCC had cirrhosis, one in the control group and one in the case group.

For the ILL cohort (n = 20 total), we reanalyzed ten non-radioembolized patients from a cohort previously published by our group and performed ILL on two additional controls in the present study (both HCC). Therefore, the control group consisted of nine patients with CRLM and three with HCC. In eight post-RL patients (cases), we performed ILL in the current study (four CRLM and four HCC). Table 2 provides detailed patient characteristics for the ILL cohort.

In the control (no RL) and case (RL) groups, five patients had evidence of cirrhosis/fibrosis and received preoperative chemotherapy (all CRLM), whereas four patients in the case group (all CRLM) received preoperative chemotherapy. The average time between the last cycle and surgery for those who received

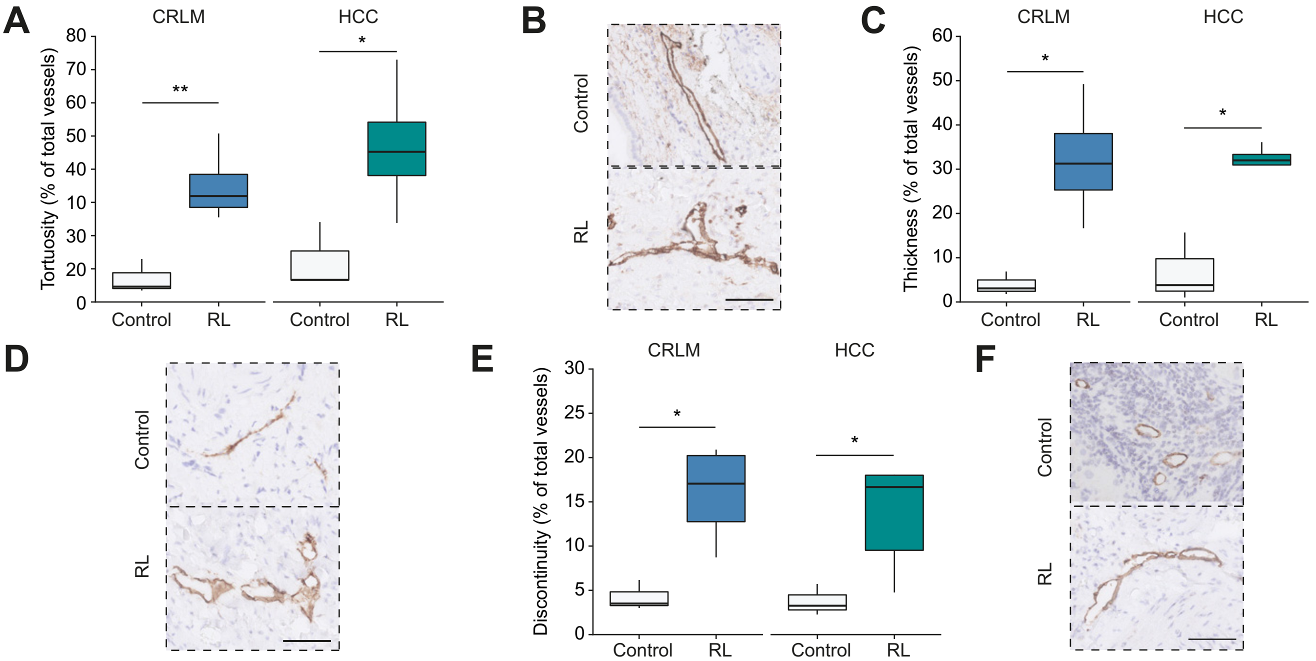

Fig. 2. Peritumor lymphatics have aberrant morphology post-RL. (A) Comparison of percentages of tortuous lymphatics in control *vs.* post-RL specimens from patients with CRLM and HCC, and representative example in (B). (C) Frequencies of thickened walls in controls *vs.* post-RL, and example in (D). (E) Discontinuity frequencies of lymphatics in control *vs.* post-RL and example in (F). CRLM control (n = 253 lymphatics; n = 3 patients), CRLM post-RL (n = 426 lymphatics; n = 4 patients), HCC control (n = 413 lymphatics; n = 3 patients) and HCC post-RL (n = 340 lymphatics; n = 5 patients). Scale bars indicate 50 μm $^{*}p <0.05$, $^{**}p <0.01$, two-tailed Student's *t* test. CRLM, colorectal liver metastases; HCC, hepatocellular carcinoma; RL, radiation lobectomy.

Table 3. Morphological characteristics of lymph vessels.

	CRLM			HCC		
	**Control (n = 3)**	**RL (n = 7)**	***p* value[§]**	**Control (n = 3)**	**RL (n = 6)**	***p* value[§]**
Tortuosity (%)	7.0 ± 3.0	35.0 ± 5.8	<0.01	12.4 ± 5.8	46.9 ± 8.2	<0.05
Thickened (%)	3.9 ± 1.5	32.1 ± 6.8	<0.05	6.9 ± 4.5	28.4 ± 4.8	<0.05
Discontinuous (%)	4.2 ± 1.0	15.9 ± 2.8	<0.05	3.8 ± 1.0	13.4 ± 2.7	<0.05
Vessel density (vessels/mm^2)	4.9 ± 1.5	10.6 ± 2.4	n.s.	16.8 ± 6.9	14.2 ± 9	n.s.
Luminal area (μm^2)	283 ± 16.9	433 ± 19.5	<0.0001	290 ± 16.9	503 ± 29.3	<0.0001

CRLM, colorectal liver metastases; HCC, hepatocellular carcinoma; RL, radiation lobectomy.
Numbers as mean ± SEM. Student's *t* test was used for tortuosity, thickness and discontinuity. Mann-Whitney *U* test was used for vessel density and luminal area.
§ Comparing non-radioembolized *vs.* radioembolized vessels.

chemo in the control group was 56 ± 52 days, while the time between RL and surgery in the case group was 97 ± 25 days.

Peritumor liver lymph vessels display abnormal morphology and signs of fluid stasis after radioembolization

We scored lymph vessels (n = 1,432) on aberrant morphology, including vessel wall tortuosity, thickness and discontinuity.[13,24–26] There were no statistically significant differences in lymph vessel morphology between non-radioembolized CRLM and non-radioembolized HCC tumors ($p >0.05$ for all morphological characteristics). However, post-RL lymph vessels, when compared to naïve or chemotherapy-treated vessels, were more often tortuous (Fig. 2A,B and Table 3, $p <0.01$ for CRLM samples, $p <0.05$ for HCC). Post-RL lymph vessel walls were also thickened (Fig. 2C,D, $p <0.05$ in both CRLM and HCC samples), and frequently displayed discontinuous, misaligned vessel walls (Fig. 2E,F, $p <0.05$ in both groups).

Dysfunctional lymph vessels cannot drain interstitial fluid, leading to stasis within the lymph vessels and thus resulting in large lumina.[27,28] Analysis of 5,695 manually annotated peritumoral lymph vessels revealed that peritumor lymph vessels of non-radioembolized CRLM and non-radioembolized HCC had similar luminal size (283 ± 16.9 μm^2 in CRLM *vs.* 290 ± 16.9 μm^2 in HCC, $p >0.05$). After radioembolization, a 1.5-fold increase in luminal size in CRLM samples was noted ($p <0.0001$, Fig. 3A and Table 3). A similar 1.7-fold increase in irradiated HCC vessels was observed ($p <0.0001$, Fig. 2B and Table 3). We frequently found the presence of vessels that were more than 25-fold larger than the median, especially in the CRLM group (Fig. 3A,B). Consistent with previous reports,[29,30] peritumoral lymph vessel density of non-radioembolized HCC tumors was higher than that of non-radioembolized CRLM tumors, albeit not statistically significant (4.9 ± 1.5 vessels/mm^2 in CRLM *vs.* 16.8 ± 6.9 vessels/mm^2 in HCC, $p >0.05$). We found no difference in the lymph vessel density

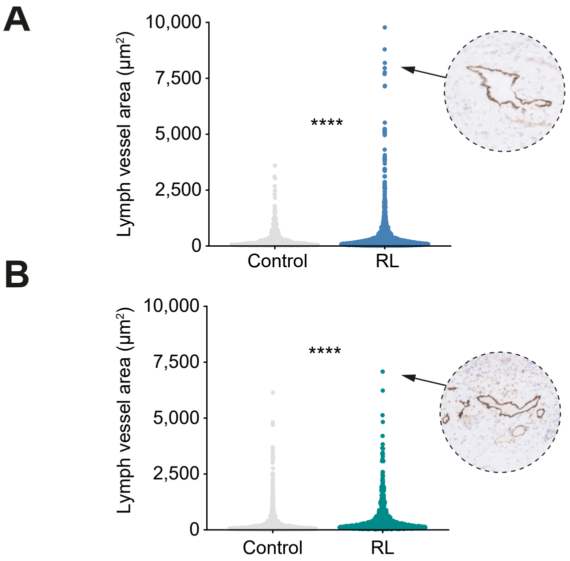

Fig. 3. Lymphatics have higher luminal size post-RL. (A). Lumen area per lymph vessel for controls with CRLM (average of 283 µm^2, n = 569 lymphatics; n = 3 patients), and after RL (433 µm^2, n = 1,773 lymphatics; n = 7 patients). (B). Per vessel lumen area in controls with HCC (290 µm^2, n = 999 lymphatics; n = 3 patients) and after RL (503 µm^2, *n* = 654 lymphatics; *n* = 6 patients). Insets indicate examples of large lymphatic vessels. ****p <10^{-4}, Mann-Whitney *U* test. CRLM, colorectal liver metastases; HCC, hepatocellular carcinoma; RL, radiation lobectomy.

between non-irradiated and irradiated samples (p = n.s. in both tumor groups).

Absence of lymphatic drainage in radioembolized livers

Next, we performed ILL in eight post-RL patients (n = 4 CRLM, n = 4 HCC). Only one patient (1/8) had blue staining of lymph node station 12 left to the hepatoduodenal ligament and another patient (1/8) had staining of a small lymph vessel right to the hepatoduodenal ligament (Fig. 4A, Table S1). We compared these patients to a cohort previously published by our group of ten patients (n = 9 CRLM, n = 1 HCC) and two additional non-radioembolized patients with HCC. In this control group, all 12 patients exhibited clear blue staining of the ipsilateral hepatic pedicle lymph nodes (12/12) and lymph vessels (12/12) upon patent blue injection (Fig. 4B, p = 1.0x10^{-4}, Fisher's exact test when compared to RL group). While these results imply radioembolization is associated with decreased lymphatic drainage, pathophysiological characteristics of the liver, such as fibrosis/cirrhosis,[31–34] steatosis,[35] and hepatitis,[36,37] have previously been reported to influence lymph vessel function. Because the small sample size precluded formal multivariate (logistic) regression analysis to account for such confounders,[38] we evaluated whether these characteristics were statistically different between radioembolized (cases) and non-radioembolized (control) patients. We found no difference between cases and controls in the prevalence of steatosis (p = 0.37, Fisher's exact test), hepatitis, fibrosis or cirrhosis (all p = 1). Moreover, there were no statistically significant difference between the groups in terms of tumor type (HCC or CRLM, p = 0.39), major or minor surgery (p = 0.17), sex (p = 0.62), age (p = 0.9) or preoperative chemotherapy status (p = 1) (Table 2 includes all potential confounders that were tested). Thus, these results suggest that radioembolization reduces the drainage of lymph from intrahepatic lymph vessels into perihilar lymph nodes and vessels.

Discussion

This study assessed lymph vessel morphology and function in post-RL livers through IHC analysis of resection specimens and ILL. Irradiated lymphatics displayed features of aberrant morphology, including tortuous, thick, and irregularly shaped vessel walls. Moreover, post-RL lymphatic vessels were found to be dysfunctional, as was indicated by their dilated lumens, suggesting lymph stasis, and the absence of lymph drainage to perihilar lymph nodes and lymph vessels.

Although this study is the first to analyze the effects of radioembolization on liver lymph vessels, radiation-induced lymph edema is a well-known side effect following radiation therapy for breast cancer.[39] In a study comparing non-irradiated and irradiated melanoma samples, radiation resulted in enlarged cutaneous lymph vessels with abnormal morphology within 4 weeks, and increased vessel density through lymphangiogenesis

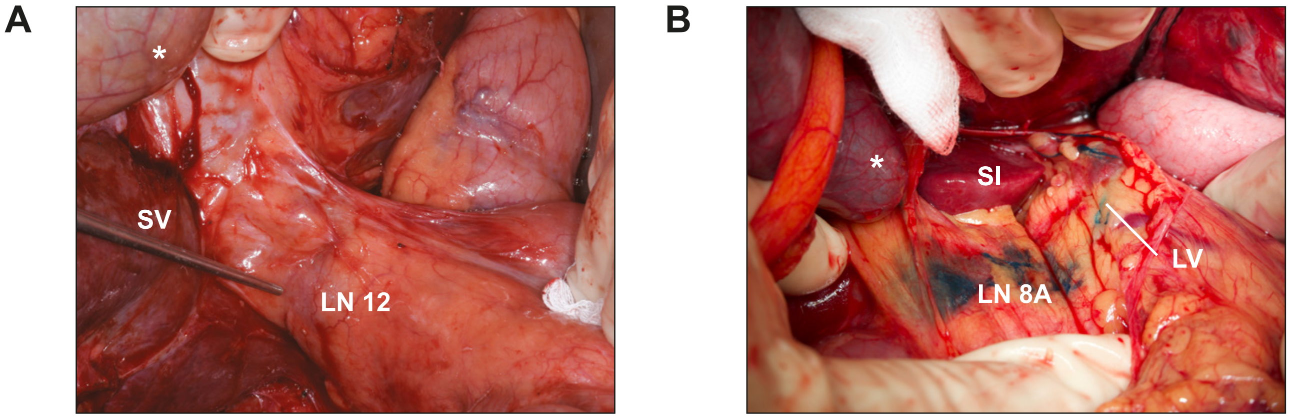

Fig. 4. Intraoperative liver lymphangiography reveals absent lymph drainage in post-RL patients. (A). After injection of patent blue dye in the parenchyma of a previously radioembolized liver, no blue staining is observed in perihilar lymph nodes or LV (depicted here is LN station 12). (B). Intraoperative liver lymphangiography in a non-irradiated control patient shows lymph vessels draining blue-dyed fluid towards LN 8A. *Gallbladder. LN, lymph node; LV, lymph vessel; RL, radiation lobectomy; S, segment.

within 1 year.[40] Moreover, it was found that radiation induces lymphatic endothelial cell apoptosis and lymphatic dysfunction through fibrosis.[13] Essentially, lymph vessels in post-RL livers resemble those seen in transgenic mouse models of defective lymph angiogenesis.[24,25] The impaired lymph drainage in the current study may thus have a dual etiology: (i) radiation-induced fibrosis, leading to blockage of lymph flow and subsequently ectatic vessels through lymph stasis, and (ii) abnormal lymph vessel morphology, either through radiation-induced apoptosis, or uncoordinated lymph angiogenesis, or both. The perihepatic lymph nodes are unlikely to block the lymph drainage: although lymph nodes may become fibrotic upon radiation damage,[26] the mean tissue penetration of the microsphere's beta emission is 2.5 mm. Hence, it may be assumed that all energy is absorbed by the liver lymph vessels within the parenchyma.

Lymphatic vessels are a frequent route for cancer dissemination, and extrahepatic metastatic spread to lymph nodes is a major negative determinant of outcome in both primary and secondary liver cancers.[14–16] Experimental work has shown that VEGF-C expressed by tumor cells increases lymphatic metastasis through several pathophysiological mechanisms including the induction of peritumor lymphatic hyperplasia, and the expression of VEGF-C has been associated with recurrence in lymph nodes and distant sites for both HCC[41] and CRLM.[16] Therefore, targeting lymph vessels represents an interesting therapeutic opportunity.[18] Moreover, numerous drugs currently in clinical trials inhibit lymphatic metastasis-promoting molecular pathways, thus underscoring the potential importance of strategies to block the dissemination of cancer cells via the lymphatics.[18,42]

From a surgical point of view, these findings underscore the rationale for using radioembolization (RL) rather than portal venous embolization as neoadjuvant therapy in the context of insufficient future liver remnant. During portal venous embolization, the portal vein of the ipsilateral, tumor-bearing liver lobe is embolized.[43] As a result, portal blood shunts towards the contralateral liver lobe and induces its growth, enabling safe resection. Portal vein embolization is associated with a low complication rate and results in future liver remnant hypertrophy within 4-6 weeks.[43] However, portal vein embolization does not treat the tumor, which is disadvantageous in patients with HCC for whom effective neoadjuvant therapy is lacking. A number of studies have demonstrated accelerated progression of existing tumors after portal vein embolization.[44–46] Conversely, approximately 30% to 50% of patients with CRLM or HCC, respectively, have a complete to partial response following radioembolization.[47,48] In a meta-analysis, patients with HCC treated with ^{90}Y radioembolization had better progression free-survival than those treated with transarterial chemoembolization.[49] We and others have recently demonstated that major hepatectomy following RL can be safely performed.[7] In light of these and the present results, we speculate that RL could control tumor growth and further spread while waiting for future liver remnant hypertrophy.

Limitations of this study include the small sample sizes. Moreover, despite the use of digital pathology software, immunohistochemical analyses remain semi-quantitative. Complete blinding was not possible due to the presence of microspheres in post-RL specimens. Finally, it was not possible to correlate lymphatic vessel dysfunction with lymphatic vessel invasion due to the scarcity of the latter event. Future studies in larger samples could reveal such events, as well as whether radioembolization is associated with reduced progression to lymph drainage-specific sites such as the perihepatic lymph nodes and lungs.

In conclusion, this study demonstrates that radioembolization impairs the function of peritumoral lymph vessels. Whether this impaired function may attenuate metastatic spread via this route warrants further translational and clinical research.

Abbreviations

^{166}Ho, holmium-166; ^{90}Y, yttrium-90; CRLM, colorectal liver metastases; HCC, hepatocellular carcinoma; IHC, immunohistochemistry; ILL, intraoperative liver lymphangiography; RL, radiation lobectomy; UMC, University Medical Center.

Financial support

This research was supported by a private fund.

Conflict of interest

Marnix Lam is a consultant for Boston Scientific and Terumo. Maarten Smits is consultant for Philips and Terumo/Quierem Medical and has served as speakers for SirTex, BTG, Swedisch Orphan Biovitrum, Terumo and Metronic. The Department of Radiology and Nuclear Medicine of the UMC Utrecht receives royalties from Quirem Medical. No other potential conflict of interest relevant to this article was reported.

Please refer to the accompanying ICMJE disclosure forms for further details.

Authors' contributions

DA designed the analysis, collected and contributed data and performed the analysis; LvdB designed the analysis, collected and contributed data; MML, MGEHL, MLJS, IQM, JdB contributed data; OK conceived and designed the analysis; IHMBR and JH conceived and designed the analysis, collected and contributed data. All authors contributed to the writing of the paper.

Data availability statement

The data supporting the findings of this study are partly available within the supplementary materials and at 10.1093/bjs/znac076. All other data are available from the corresponding author upon reasonable request.

Acknowledgements

The authors thank Domenico Castigliego and colleagues of the UMCU tissue facility for continuous support and Niek Peters, Esther Strating and Susanne van Schelven for scientific discussions and practical help.

Supplementary data

Supplementary data to this article can be found online at https://doi.org/10.1016/j.jhepr.2023.100981.

References

Author names in bold designate shared co-first authorship

[1] **Gans JH**, Lipman J, Golowa Y, et al. Hepatic cancers overview: surgical and chemotherapeutic options, how do Y-90 microspheres fit in? Semin Nucl Med 2019 May;49(3):170–181.
[2] **Tchelebi L**, Sharma NK. Selective internal radiation therapy in the multidisciplinary management of liver metastases from colorectal carcinoma. Semin Nucl Med 2019 May;49(3):182–188.
[3] **Reig M**, Forner A, Rimola J, et al. BCLC strategy for prognosis prediction and treatment recommendation: the 2022 update. J Hepatol 2022 Mar;76(3):681–693.

[4] **Gaba RC**, Lewandowski RJ, Kulik LM, et al. Radiation lobectomy: preliminary findings of hepatic volumetric response to lobar yttrium-90 radioembolization. Ann Surg Oncol 2009 Jun 9;16(6):1587–1596.
[5] **Vouche M**, Lewandowski RJ, Atassi R, et al. Radiation lobectomy: time-dependent analysis of future liver remnant volume in unresectable liver cancer as a bridge to resection. J Hepatol 2013 Nov;59(5):1029–1036.
[6] **Lewandowski RJ**, Donahue L, Chokechanachaisakul A, et al. 90Y Radiation lobectomy: outcomes following surgical resection in patients with hepatic tumors and small future liver remnant volumes. J Surg Oncol 2016;114(1):99–105.
[7] **Andel D**, Dassen MG, Reinders-Hut MTM, et al. Surgical outcomes of major hepatectomy following "radiation lobectomy" for hepatic malignancies and insufficiently functional future liver remnant: initial experience. Br J Surg 2020 Oct 14;107(12):e609–e610.
[8] **Barker HE**, Paget JTE, Khan AA, et al. The tumour microenvironment after radiotherapy: mechanisms of resistance and recurrence. Nat Rev Cancer 2015 Jul 24;15(7):409–425.
[9] **Mantoni TS**, Lunardi S, Al-Assar O, et al. Pancreatic stellate cells radioprotect pancreatic cancer cells through β1-integrin signaling. Cancer Res 2011 May 15;71(10):3453–3458.
[10] **Yarnold J**, Vozenin Brotons MC. Pathogenetic mechanisms in radiation fibrosis. Radiother Oncol 2010 Oct;97(1):149–161.
[11] **Chew V**, Lee YH, Pan L, et al. Immune activation underlies a sustained clinical response to Yttrium-90 radioembolisation in hepatocellular carcinoma. Gut 2019 Feb;68(2):335–346.
[12] **Langley R**, Bump E, Quartuccio S, et al. Radiation-induced apoptosis in microvascular endothelial cells. Br J Cancer 1997 Mar;75(5):666–672.
[13] **Avraham T**, Yan A, Zampell JC, et al. Radiation therapy causes loss of dermal lymphatic vessels and interferes with lymphatic function by TGF-β1-mediated tissue fibrosis. Am J Physiology-Cell Physiol 2010 Sep;299(3):C589–C605.
[14] **Hodgson R**, Sethi H, Ling AH, et al. Combined hepatectomy and hepatic pedicle lymphadenectomy in colorectal liver metastases is justified. HPB 2017 Jun;19(6):525–529.
[15] **Grobmyer SR**, Wang L, Gonen M, et al. Perihepatic lymph node assessment in patients undergoing partial hepatectomy for malignancy. Ann Surg 2006 Aug;244(2):260–264.
[16] **Vellinga TT**, Kranenburg O, Frenkel N, et al. Lymphangiogenic gene expression is associated with lymph node recurrence and poor prognosis after partial hepatectomy for colorectal liver metastasis. Ann Surg 2017 Nov;266(5):765–771.
[17] **Tammela T**, Saaristo A, Holopainen T, et al. Photodynamic ablation of lymphatic vessels and intralymphatic cancer cells prevents metastasis. Sci Transl Med 2011 Feb 9;(69):3.
[18] **Roy S**, Banerjee P, Ekser B, et al. Targeting lymphangiogenesis and lymph node metastasis in liver cancer. Am J Pathol 2021 Dec;191(12):2052–2063.
[19] **van den Bent L**, Frenkel NC, Poghosyan S, et al. Liver lymphatic drainage patterns follow segmental anatomy. Br J Surg 2022 May 16;109(6):559–560.
[20] **Frenkel NC**, Poghosyan S, Verheem A, et al. Liver lymphatic drainage patterns follow segmental anatomy in a murine model. Sci Rep 2020 Dec 11;10(1):21808.
[21] **Weber M**, Lam M, Chiesa C, et al. EANM procedure guideline for the treatment of liver cancer and liver metastases with intra-arterial radioactive compounds. Eur J Nucl Med Mol Imaging 2022 Apr 11;49(5):1682–1699.
[22] **de Graaf W**, van Lienden KP, Dinant S, et al. Assessment of future remnant liver function using hepatobiliary scintigraphy in patients undergoing major liver resection. J Gastrointest Surg 2010;14(2):369–378.
[23] **Bullitt E**, Gerig G, Pizer SM, et al. Measuring tortuosity of the intracerebral vasculature from MRA images. IEEE Trans Med Imaging 2003 Sep;22(9):1163–1171.
[24] **Petrova TV**, Karpanen T, Norrmén C, et al. Defective valves and abnormal mural cell recruitment underlie lymphatic vascular failure in lymphedema distichiasis. Nat Med 2004 Sep 22;10(9):974–981.
[25] **Ribatti D**. Transgenic mouse models of angiogenesis and lymphangiogenesis. Int Rev Cel Mol Biol 2008 Jan 1;266:1–35.
[26] **Allam O**, Park KE, Chandler L, et al. The impact of radiation on lymphedema: a review of the literature. Gland Surg 2020 Apr;9(2):596–602.
[27] **Barone V**, Borghini A, Tedone Clemente E, et al. New insights into the pathophysiology of primary and secondary lymphedema: histopathological studies on human lymphatic collecting vessels. Lymphat Res Biol 2020 Dec 1;18(6):502–509.
[28] **Liao XB**, Tang WP, Zhang QM, et al. Morphological analysis of lymph vessels and capillaries in gastric carcinoma. World J Gastroenterol 2011;3(2):90.
[29] **Schoppmann A**, Tamandl D, Herberger B, et al. Comparison of lymphangiogenesis between primary colorectal cancer and corresponding liver metastases. Anticancer Res 2011 Dec;31(12):4605–4611.
[30] **Thelen A**, Jonas S, Benckert C, et al. Tumor-associated lymphangiogenesis correlates with prognosis after resection of human hepatocellular carcinoma. Ann Surg Oncol 2009 May 18;16(5):1222–1230.
[31] **Dumont AE**, Mulholland JH. Flow rate and composition of thoracic-duct lymph in patients with cirrhosis. New Engl J Med 1960 Sep 8;263(10):471–474.
[32] **Barrowman JA**, Granger DN. Effects of experimental cirrhosis on splanchnic microvascular fluid and solute exchange in the rat. Gastroenterology 1984 Jul;87(1):165–172.
[33] **Vollmar B**, Wolf B, Siegmund S, et al. Lymph vessel expansion and function in the development of hepatic fibrosis and cirrhosis. Am J Pathol 1997 Jul;151(1):169–175.
[34] **Yamauchi Y**. Morphometric analysis of lymphatic vessels in primary biliary cirrhosis. Hepatol Res 2002 Oct;24(2):107–113.
[35] **Burchill MA**, Finlon JM, Goldberg AR, et al. Oxidized low-density lipoprotein drives dysfunction of the liver lymphatic system. Cell Mol Gastroenterol Hepatol 2021;11(2):573–595.
[36] **Tamburini BAJ**, Finlon JM, Gillen AE, et al. Chronic liver disease in humans causes expansion and differentiation of liver lymphatic endothelial cells. Front Immunol 2019;10:1036.
[37] **Yamauchi Y**, Michitaka K, Onji M. Morphometric analysis of lymphatic and blood vessels in human chronic viral liver diseases. Am J Pathol 1998 Oct;153(4):1131–1137.
[38] **Concato J**, Peduzzi P, Holford TR, et al. Importance of events per independent variable in proportional hazards analysis I. Background, goals, and general strategy. J Clin Epidemiol 1995 Dec;48(12):1495–1501.
[39] **Warren LEG**, Miller CL, Horick N, et al. The impact of radiation therapy on the risk of lymphedema after treatment for breast cancer: a prospective cohort study. Int J Radiat Oncology*Biology*Physics 2014 Mar;88(3):565–571.
[40] **Jackowski S**, Janusch M, Fiedler E, et al. Radiogenic lymphangiogenesis in the skin. Am J Pathol 2007 Jul 1;171(1):338–348.
[41] **Yamaguchi R**, Yano H, Nakashima O, et al. Expression of vascular endothelial growth factor-C in human hepatocellular carcinoma. J Gastroenterol Hepatol 2006 Jan;21(1):152–160.
[42] **Dieterich LC**, Detmar M. Tumor lymphangiogenesis and new drug development. Adv Drug Deliv Rev 2016 Apr;99:148–160.
[43] **Madoff DC**, Gaba RC, Weber CN, et al. Portal venous interventions: state of the art. Radiology 2016 Feb;278(2):333–353.
[44] **Hoekstra LT**, van Lienden KP, Doets A, et al. Tumor progression after preoperative portal vein embolization. Ann Surg 2012 Nov;256(5):812–818.
[45] **Pamecha V**, Levene A, Grillo F, et al. Effect of portal vein embolisation on the growth rate of colorectal liver metastases. Br J Cancer 2009 Feb 10;100(4):617–622.
[46] **Hayashi S**, Baba Y, Ueno K, et al. Acceleration of primary liver tumor growth rate in embolized hepatic lobe after portal vein embolization. Acta Radiol 2007 Sep 30;48(7):721–727.
[47] **Alsultan AA**, van Roekel C, Barentsz MW, et al. Dose–response and dose–toxicity relationships for glass ^{90} Y radioembolization in patients with liver metastases from colorectal cancer. J Nucl Med 2021 Nov;62(11):1616–1623.
[48] **Kappadath SC**, Mikell J, Balagopal A, et al. Hepatocellular carcinoma tumor dose response after 90Y-radioembolization with glass microspheres using 90Y-SPECT/CT-Based voxel dosimetry. Int J Radiat Oncology*Biology*Physics 2018 Oct;102(2):451–461.
[49] **Facciorusso A**, Serviddio G, Muscatiello N. Transarterial radioembolization *vs* chemoembolization for hepatocarcinoma patients: a systematic review and meta-analysis. World J Hepatol 2016;8(18):770.

Journal of Hepatology, Volume *6*

Supplemental information

^{90}Y-/^{166}Ho- `Radiation lobectomy' for liver tumors induces abnormal morphology and impaired drainage of peritumor lymphatics

Daan Andel, Lotte van den Bent, Marnix Gerard Ernest Hendrik Lam, Maarten Leonard Johannes Smits, Isaac Quintus Molenaar, Joep de Bruijne, Miangela Marie Laclé, Onno Kranenburg, Inne Hildbrand Max Borel Rinkes, and Jeroen Hagendoorn

^{90}Y-/^{166}Ho- 'Radiation lobectomy' for liver tumors induces abnormal morphology and impaired drainage of peritumor lymphatics

Daan Andel, Lotte van den Bent, Marnix Gerard Ernest Hendrik Lam, Maarten Leonard Johannes Smits, Isaac Quintus Molenaar, Joep de Bruijne, Miangela Marie Lacle, Onno Kranenburg, Inne Hildbrand Max Borel Rinkes, Jeroen Hagendoorn

Table of contents

Supplementary data file 1……………………………………………..…………..………2

Fig. S1………………………………………………………………………………………5

Fig. S2………………………………………………………………………………………6

Table S1…………………………………………………………………………………… 7

Supplementary reference…………………………………………………………….…….. 8

Supplementary data file 1

LYMPHANGIOGRAFIE / LIVER RESECTION – CRF

Hospital: ………..
Patient number: ………..
OK: date: ………..

Surgeon:

1th assistent:

Indication (tumor type):
Primary tumor in situ (CRLM):
Preop. Chemo:
Preop. Y90:
Procedure performed (wig/segments etc.):
Open / robot:
Injection Patent Blue in segment:
Total ml injected (number of fractions):
Lymph vessels on capsule blue (accidental): yes/no
Time injection untill blue staining of lymph vessels in ligament or nodes (minutes):
Video or photos procedure:

Nodes

**Yes* No-**	**Lymph station**	**After how many minutes.***	**Description**	**PA vial #**
-	12right		Right side ligament (along bile duct/porta)	
-	12left		Left side ligament (along a hep sinistra)	
-	13		Head of pancrease/base of ligament	
-	8A		A hep communis	
-	9		Truncus	
-	7		Omentus minus	
	~		D. cysticus	

-	~		Suprahepatische VCI (left/right)	
	~		Other, nl.:	

***Total minutes between injection and first blue staining of node**

Lymph vessels visible (blue discoloration)

Yes* No-	Location
-	Right side ligament
-	Left side ligament
-	In parenchyma during transection
-	Along right / middle / left hepatic vein
-	Diaphragm
-	Omentum minus

Arterial anatomy

Normal yes/no

If anatomical variant, describe:

Replaced/accessory AHD from the AMS

Replaced/accessory a. hep. sinistra from the gastrica sinistra

Other, namely………

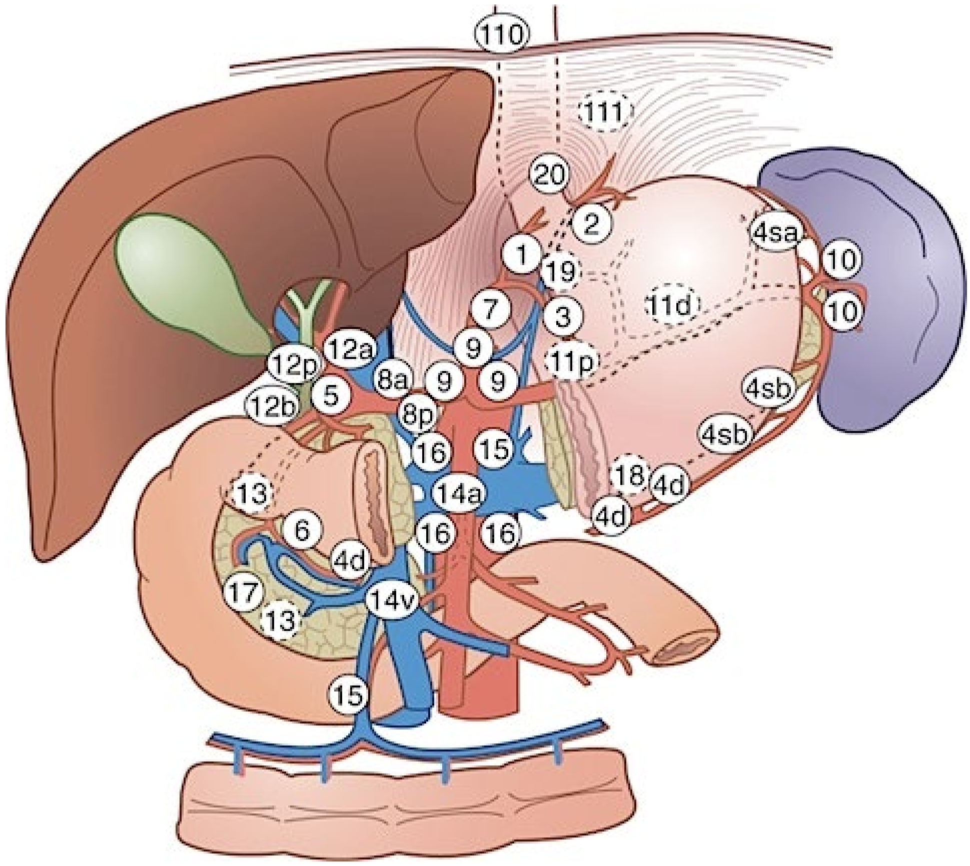
110
111
20
2
4sa
10
1
19
10
7
3
11d
12a
9
12p
11p
8a
9
9
4sb
5
12b
8p
4sb
16
15
18
4d
13
14a
4d
6
16
16
4d
17
14v
13
15

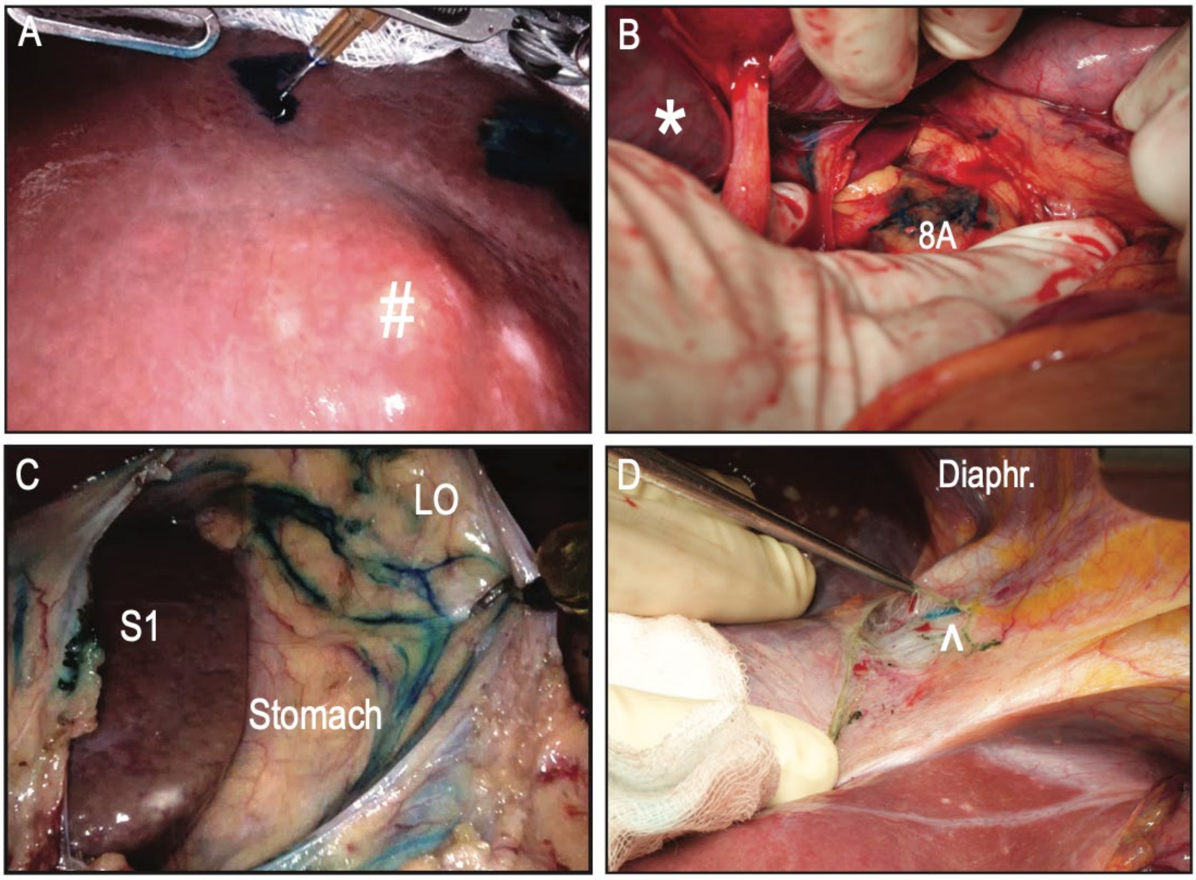

Fig. S1. Procedures of intraoperative liver lymphangiography
(A). Injection if Patent blue dye 2 cm away from the tumor (#). (B). After injection of dye, the perihilar region is inspected for blue staining. Here, lymph node station 8A is depicted. (C). Visualisation of the lesser omentum, containing blue stained lymphatic vessels. (D). Blue-stained lymphatic vessels (arrow) are seen along the suprahepatic inferior vena cava. Note that the figures are not from the same procedure. The procedures were performed in patients with non-radioembolized livers.

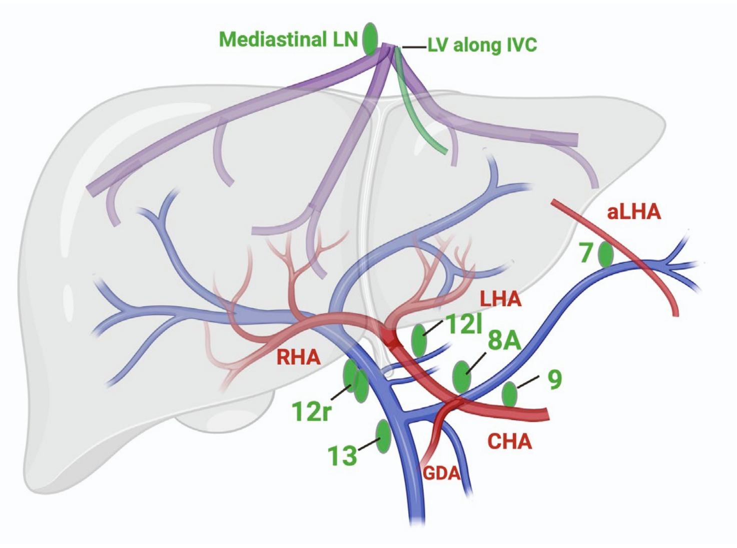

Fig. S2. Schematic representation of perihepatic lymph nodes. Green circles represent lymph nodes. Blue and purple vessels represent portal and hepatic veins, respectively. Red vessels represent hepatic arteries. aLHA: accessory/replaced left hepatic artery, CHA: common hepatic artery, GDA: gastroduodenal artery, IVC: inferior vena cava, LHA: left hepatic artery, LV: lymph vessel, RHA: right hepatic artery.

Table S1. Intraoperative liver lymphangiography details

	ILL1	ILL2	ILL3	ILL4	ILL5	ILL6	ILL7	ILL8	ILL9	ILL10
**Tumour type**	CRLM	CRLM	CRLM	CRLM	HCC	HCC	HCC	HCC	HCC	HCC
**Case/control**	Case	Case	Case	Case	Case	Case	Case	Case	Control	Control
**Injection Patent blue**										
Location	S5	S6/S7	S7	S2/S3	S6	S7	S8	S6	S6/7	S4A
Volume (mL)	2	4	3	3	3	2	2	3	4	4
Time to staining (min)	5	NA	NA	2	NA	NA	NA	NA	6	
**LN station / location**										
12 right	-	-	-	-	-	-	-	-	-	X
12 left	-	-	-	X	-	-	-	-	-	X
13	-	-	-	-	-	-	-	-	-	
8A	-	-	-	-	-	-	-	-	X	X
9	-	-	-	-	-	-	-	-	-	-
7	-	-	-	-	-	-	-	-	-	-
D. cysticus	-	-	-	-	-	-	-	-	-	-
Suprahepatic VCI right	-	-	-	-	-	-	-	-	-	-
Suprahepatic VCI left	-	-	-	-	-	-	-	-	-	-
Other	-	-	-	-	-	-	-	-	-	-
**LV location**										
Hepatoduodenal lig. Right	X	-	-	-	-	-	-	-	X	-
Hepatoduodenal lig. Left	-	-	-	-	-	-	-	-	X	X
Parenchymal	-	-	-	-	-	-	-	-	-	-
Left hepatic vein	-	-	-	-	-	-	-	-	-	-
Middle hepatic vein	-	-	-	-	-	-	-	-	-	-
Right hepatic vein	-	-	-	-	-	-	-	-	-	-
Diaphragm	-	-	-	-	-	-	-	-	-	-
Omentum minus	-	-	-	-	-	-	-	-	-	-
Other	-	-	-	-	-	-	-	-	-	-

'X' indicates blue staining was observed, CRLM: colorectal liver metastases, HCC: hepatocellular carcinoma, LN: lymph node, LV: lymph vessel. Only those patients that were assessed in this study were included. All other (control) patients are described in *'van den Bent et al., BJS, 2022'* (1).

Supplementary reference

1. van den Bent L, Frenkel NC, Poghosyan S, Molenaar IQ, Padera TP, Kranenburg O, et al. Liver lymphatic drainage patterns follow segmental anatomy. British Journal of Surgery. 2022 May 16;109(6):559–60.
